# Improving outcomeS for Women diagnosed with early breast cancer through adhErence to adjuvant Endocrine Therapy (SWEET): study protocol for a pragmatic randomised control trial of a patient-centred intervention to improve adherence to endocrine therapy in early breast cancer

**DOI:** 10.1186/s13063-025-09056-6

**Published:** 2025-11-26

**Authors:** Lucy McGeagh, Alice Walker, Louise Hiller, Janet Dunn, Mary Wells, Peter Donnelly, Andrew Wardley, Jane Wolstenholme, Robert Horne, Sarah-Jane Stewart, Jo Brett, Caitriona Cahir, Deborah Fenlon, Jan Rose, Lesley Turner, Lyndsay Hughes, Adam Todd, Brian Nicholson, Phil Mawson, Ruth Norris, Sue Thompson, Helen Dakin, Raegan Barrows, Sally Kum, Mark Turner, Farah Rehman, Henry Cain, Eila Watson, Linda Sharp, SWEET Research Team, Alex King, Victoria Harmer, Nicky Levitt, Anna Mann, Colin McCowan, Zoe Moon, Mia Porteous, Samuel G. Smith

**Affiliations:** 1 Oxford Institute of Applied Health Research, Oxford Brookes University, Oxford, UK; 2 Warwick Clinical Trials Unit, Warwick Medical School, University of Warwick, Coventry, UK; 3 Imperial College Healthcare NHS Trust, London, UK; 4 Imperial College London, London, UK; 5 Torbay and South Devon NHS Foundation Trust, Torquay, UK; 6 Outreach Research & Innovation Group, Manchester, UK; 7 Health Economics Research Centre, Nuffield Department of Population Health, University of Oxford, Oxford, UK; 8 Research Department of Practice and Policy, UCL School of Pharmacy, University College London, London, UK; 9 School of Population Health, RCSI University of Medicine and Health Sciences, Dublin, Ireland; 10 Professor Emerita, Faculty Of Medicine, Health and Life Science, Swansea University, Swansea, UK; 11 Independent Cancer Patients’ Voice, UK and Cancer Research Advocates Forum-UK, London, UK; 12 Kings College London, London, UK; 13 School of Pharmacy, Newcastle University, Newcastle, UK; 14 Department of Primary Care Health Sciences, University of Oxford, Oxford, UK; 15 Translational and Clinical Research Institute, Newcastle University, Newcastle, UK; 16 Population Health Sciences Institute, Newcastle University, Newcastle, UK; 17 Breast Cancer Now, London, UK; 18 Research Software Engineering, Newcastle University, Newcastle, UK; 19 Newcastle Hospitals NHS Foundation Trust, Newcastle, UK; 20 Oxford University Hospitals NHS Foundation Trust, Oxford, United Kingdom; 21 School of Medicine, University of St Andrews, St Andrews, United Kingdom; 22 22Leeds Institute of Health Sciences, School of Medicine, University of Leeds, Leeds, United Kingdom

**Keywords:** Breast cancer, Endocrine therapy, Adherence, Quality-of-life, Intervention, Self-management, Randomised control trial

## Abstract

**Background:**

At least 5 years of adjuvant endocrine therapy substantially reduces risks of recurrence and mortality in oestrogen-receptor positive early breast cancer. However, adherence to endocrine therapy is sub-optimal; poor adherence is associated with higher risks of recurrence and death from breast cancer, worse cancer-specific health-related quality-of-life, and increased healthcare costs. The SWEET randomised control trial aims to evaluate effectiveness and cost-effectiveness of the HT&Me intervention in reducing poor adherence to adjuvant endocrine therapy and improve cancer-specific health-related quality-of-life in women with oestrogen-receptor positive early breast cancer.

**Methods:**

This is a UK based, pragmatic, open label randomised control trial. Participants (stages 1–3 oestrogen-receptor positive breast cancer, completed surgery, within 14 weeks of first endocrine therapy prescription; *n* = 1460) complete a baseline questionnaire, and are randomised to the HT&Me intervention plus usual care, or usual care alone. The HT&Me intervention is evidence-based, theory-informed and patient-centred. It consists of viewing an animation, two consultations with a SWEET study practitioner (a health care professional trained in delivering the intervention) approximately 3 months apart, access to the interactive HT&Me web-app for the 18 months, and regular monthly nudges. All participants complete follow-up questionnaires at 6, 12, and 18 months. A multi-method process evaluation will be conducted involving quantitative analysis exploring mechanisms of action of the intervention, and qualitative interviews with a sample of participants and health care professionals involved in the trial. Primary endpoints are adjuvant endocrine therapy adherence (combined self-report (Medication Adherence Report Scale) and Proportion of Days Covered calculated from prescription encashment records) and cancer-specific health-related quality-of-life (Functional Assessment of Cancer Therapy Scale- General). Secondary endpoints are adjuvant endocrine therapy-specific health-related quality-of-life and within-trial cost-utility analysis which will evaluate cost-effectiveness.

**Discussion:**

The SWEET trial seeks to address a significant issue affecting the growing population of breast cancer survivors: poor adherence to adjuvant endocrine therapy. Challenges addressed and resolved within the protocol include the following: capacity at sites to deliver the intervention; variations in breast cancer services nationally; and measuring adherence. This trial has potential to improve quality of life and adherence to endocrine therapy; reducing numbers of recurrences and breast cancer deaths, benefiting women, their families and the health service.

**Trial registration:**

ISRCTN Number: ISRCTN24852890 registered on 02.08.2023.

**Supplementary Information:**

The online version contains supplementary material available at 10.1186/s13063-025-09056-6.

## Administrative information

Note: the numbers in curly brackets in this protocol refer to SPIRIT checklist item numbers. The order of the items has been modified to group similar items (see http://www.equator-network.org/reporting-guidelines/spirit-2013-statement-defining-standard-protocol-items-for-clinical-trials/).

**Table:** 

Title {1}	Improving outcomeS for Women diagnosed with early breast cancer through adhErence to adjuvant Endocrine Therapy (SWEET): study protocol for a pragmatic Randomised Control Trial of a patient-centred intervention to improve adherence to endocrine therapy in early breast cancer
Trial registration {2a and 2b}.	ISRCTN Number: ISRCTN24852890
Protocol version {3}	08.04.2024 Version 4.0
Funding {4}	This article presents independent research funded by the National Institute for Health and Care Research (NIHR) under the Programme Grants for Applied Research programme [NIHR200098]. The views expressed in this article are those of the authors and not necessarily those of the NHS, the NIHR or the Department of Health.
Author details {5a}	*Lucy McGeagh*^*1*^*^, Alice Walker*^*2*^*^ Louise Hiller*^*2*^*, Janet Dunn*^*2*^*, Mary Wells*^*3,4*^*, Peter Donnelly*^*5*^*, Andrew Wardley*^*6*^*, Jane Wolstenholme*^*7*^*, Robert Horne*^*8*^*, Sarah-Jane Stewart*^*8*^*, Jo Brett*^*1*^*, Caitriona Cahir*^*9*^*, Deborah Fenlon*^*10*^*, Jan Rose*^*11*^*, Lesley Turner*^*11*^*, Lyndsay Hughes*^*12*^*, Adam Todd*^*13*^*, Brian Nicholson*^*14*^*, Phil Mawson*^*15*^*, Ruth Norris*^*16*^*, Sue Thompson*^*16*^*, Helen Dakin*^*7*^*, Raegan Barrows*^*2*^*, Sally Kum*^*17*^*, Mark Turner*^*18*^*, Farah Rehman*^*3*^*, Henry Cain*^*19*^*,, Eila Watson*^1^* and Linda Sharp^16^* + *, on behalf of the SWEET Research Team In addition to the named authors, the SWEET Research*Team include Alex King^3^, Victoria Harmer^3^, Nicky Levitt^20^, Anna Mann^2^, Colin McCowan^21^, Zoe Moon^8^, Mia Porteous^15^, Samuel G. Smith^22^. ^Authors contributed equally and should be considered joint lead authors *Authors contributed equally and should be considered joint senior authors ^1^Oxford Institute of Applied Health Research, Oxford Brookes University, Oxford, UK ^2^Warwick Clinical Trials Unit, Warwick Medical School, University of Warwick, Warwick, UK ^3^Imperial College Healthcare NHS Trust, London, UK ^4^Imperial College London, London, UK ^5^South Devon Healthcare NHS Foundation Trust, Torbay, UK ^6^Outreach Research & Innovation Group, Manchester, UK ^7^Health Economics Research Centre, Nuffield Department of Population Health, University of Oxford, Oxford, UK ^8^Research Department of Practice and Policy, UCL School of Pharmacy, University College London, London, UK ^9^School of Population Health, RCSI University of Medicine and Health Sciences, Dublin, Ireland ^10^Professor Emerita, Faculty Of Medicine, Health and Life Science, Swansea University, Swansea, UK ^11^Independent Cancer Patients’ Voice, UK ^12^Kings College London, London, UK ^13^School of Pharmacy, Newcastle University, Newcastle, UK ^14^Department of Primary Care Health Sciences, University of Oxford, Oxford, UK ^15^Translational and Clinical Research Institute, Newcastle University, Newcastle, UK ^16^Population Health Sciences Institute, Newcastle University, Newcastle, UK ^17^Breast Cancer Now, UK ^18^Research Software Engineering, Newcastle University, Newcastle, UK ^19^Newcastle Hospitals NHS Foundation Trust, Newcastle, UK ^20^School of Medicine, University of St Andrews, St Andrews, UK ^21^School of Medicine, University of Leeds, Leeds, UK ^22^Oxford University Hospitals NHS Foundation Trust, Oxford, UK
Name and contact information for the trial sponsor {5b}	Newcastle upon Tyne Hospitals NHS Foundation Trust Email: tnu-tr.sponsormanagement@nhs.net
Role of sponsor {5c}	The research is sponsored by Newcastle upon Tyne Hospitals NHS Foundation Trust Email: tnu-tr.sponsormanagement@nhs.net The views expressed in this article are those of the authors and not necessarily those of the sponsor. The sponsor had no role in the study design; collection, management, analysis, and interpretation of data; writing of the report; and the decision to submit the report for publication.

## Introduction

### Background and rationale {6a}

Breast cancer is the most common cancer in women worldwide, with 2.3 million women diagnosed in 2022 [1]. Although survival rates are high, in 2022, an estimated 670,000 women died from the disease globally [1]. Approximately 80% of women with breast cancer have oestrogen receptor positive (ER + ve) disease; this means that oestrogen can stimulate the cells of these tumours to grow. Most of these women undergo surgery and, if needed, radiotherapy and/or chemotherapy. Post-surgery, the majority are recommended endocrine therapy, also known as hormone therapy, in the form of a daily tamoxifen or aromatase inhibitor tablet. Adjuvant endocrine therapy (AET) significantly reduces the risks of recurrence, death from breast cancer, and death from any cause when taken for 5 years [2–4], with recent evidence showing that longer use confers further benefit [5, 6]. In light of this, guidance recommends extending AET use, for some groups of women, beyond 5 years [4, 7].

Despite these recognised benefits, many women do not take AET as recommended. Between 20 and 40% of women have suboptimal implementation of AET (taking less than the recommended dose by, for example, skipping tablets) [8–14]. Early discontinuation (stopping before the end of the recommended treatment period) occurs in around 20% by 2 years and up to 50% by 5 years [8, 11, 12]. In addition, women who display suboptimal AET implementation in the first year of therapy are more likely to discontinue therapy early [15]. AET non-adherence is associated with up to threefold increased risks of breast cancer recurrence and mortality [8–13], significantly poorer cancer-specific health-related quality-of-life (HRQoL) [13], and significantly higher health service costs [16].

Our recent umbrella review highlights the many and varied determinants of AET adherence in women with breast cancer [17]. Research indicates that beliefs about AET, including doubts about the need to take AET both every day and long-term, and misconceptions about risk of recurrence, frequently predict non-adherence [17]. Also important to adherence are concerns about AET, particularly around side-effects commonly attributed to the medication (e.g. severe hot flushes, joint pain, weight gain, depression, osteoporotic fractures) and potential long-term adverse effects (e.g. cardiovascular events), and more general negative attitudes towards long-term medication taking [17]. Other concerns include desire to move on and leave cancer behind. Motivation to take AET is important, as is capability: poor medication management techniques and forgetfulness also relate to non-adherence [17].

Interventions to improve AET adherence have been developed [18]. However, despite the complexity in determinants of adherence, many early interventions were based simply on education and increasing knowledge, and were often ineffective, perhaps unsurprisingly, given that improving knowledge alone is unlikely to be sufficient for behaviour change [19]. Other interventions have had varied success; in settings where patients contribute financially to treatment costs, interventions which reduce AET costs consistently improve adherence, whereas medication reminders, communication, and psychological/coping strategies showed varied effectiveness [18]. In addition to this, past interventions have not always been patient-centred, with an additional lack of evidence on cost-effectiveness of interventions generally in cancer self-management interventions [20].

A further limitation of some past interventions is a lack of a strong theoretical basis, despite guidance highlighting the importance of this when developing complex interventions [21]. The National Institute for Health and Care Excellence (NICE) Medicines Adherence Guidelines [22] recommends the Perceptions and Practicalities Approach (PaPA) as an overarching framework for developing, and providing, adherence support [23]. The PaPA takes a ‘no-blame’ approach to non-adherence, addressing both perceptual (e.g. beliefs about the necessity of treatment and concerns about taking it [24]) and practical barriers to adherence, acting through patients’ motivation and ability to start and continue with treatment [16]. We have demonstrated the importance of the components of PaPA to AET adherence [25]. To our knowledge, no previously published AET interventions have been developed using the PaPA framework.

The SWEET research programme has developed a patient-centred, evidence-based, theoretically-informed (PaPA), intervention to support women with AET adherence and to improve QoL—called HT&Me [26]. A feasibility study has been conducted, demonstrating the feasibility of recruitment, delivery of the intervention, collection of outcome measures, as well as acceptability and potential usefulness of the intervention to women [27]. This paper reports the protocol for the randomised control trial (RCT) which aims to evaluate the effectiveness and cost-effectiveness of the HT&Me intervention in reducing poor adherence and improving cancer-specific HRQoL. This trial has the potential to both improve HRQoL in women with breast cancer and reduce numbers of recurrences and breast cancer deaths, by improving adherence to AET. It will thus benefit women, their families and the UK publicly funded National Health Service (NHS).

### Objectives {7}

The trial objective is to determine the clinical and cost-effectiveness of a tailored, supported self-management intervention (HT&Me) in reducing poor adherence to AET and improving cancer-specific HRQoL.

#### Outcomes

The primary outcomes are AET adherence and cancer specific HRQoL. At the request of the funders National Institute for Health and Care Research (NIHR PGfAR), the trial has two primary outcomes. We have powered the trial to detect differences in these joint primary outcomes (poor adherence and cancer-specific HRQoL). The ‘decision-making framework’ which we will adopt is that the intervention will be considered efficacious if it has effects on both outcomes [28, 29]; therefore, no adjustments will be made to control the Type I error rate [30], which will be 0.05 throughout. Secondary outcomes are AET-specific HRQoL and cost-effectiveness. Details of how these are being measured and other outcomes or postulated mediators are shown in Table 1.

**Table 1:** Primary, secondary and other outcomes, postulated mediators and instrument/source of information

Outcome	Instrument/source of information
***Primary outcomes***	
AET adherence	Combined self-report (Medication Adherence Report Scale (MARS-5)) [31] and Proportion of Days Covered (PDC) calculated from prescription encashment records
Cancer-specific HRQoL	Functional Assessment of Cancer Therapy Scale-General (FACT-G) [32]
***Secondary outcomes***	
AET-specific HRQoL	Breast Cancer Trialist Prevention checklist (BCPT) [33]
Cost-effectiveness	Within-trial cost per quality-adjusted life year (QALY) QALYs will be estimated as the area under the curve for EQ-5D-5L [34]
***Additional outcomes***	
Aspects of adherence: Extent of adherence Suboptimal implementation Non-persistence	Prescription records (PDC continuous measure) Self-report (MARS-5 continuous measure; how frequently take medication on average*; how frequently taken medication in last 7 days*) >180 days gap in prescriptions and self-report*
HRQoL Cost	EQ-5D-5L [34] Resource use and cost to the NHS, patients and society (collected using patient-completed resource use questionnaire and AET prescription data)
***Postulated mediators (examples)***	
Self-efficacy for taking AET	Questions adapted from validated measures [15, 35]
Self-efficacy	Self-efficacy for coping with symptoms, adapted from validated measures [36]
Cancer-related distress	FACT-G emotional well-being subscale [32]
Medication and illness beliefs	Illness Perception Questionnaire for Breast Cancer Scale (IPQ-BCS) [37]; Beliefs about Medicines Questionnaire- Adjuvant Endocrine Therapy (BMQ-AET) [38]
Satisfaction with information about AET	Satisfaction with Information about Medicines Scale (SIMS) [39]
Physical activity	Godin-Shephard leisure-time physical activity questionnaire [40]
Practical barriers to adherence and increased self-efficacy for managing treatment	Cancer Survivor Self-Efficacy Scale (CS-SES), adapted from validated measures [41]

### Trial design {8}

This is a multi-centre, unblinded, pragmatic, superiority RCT of the HT&Me intervention plus usual care versus usual care alone in (i) reducing poor AET adherence and (ii) improving cancer-specific HRQoL.

#### Public and patient involvement (PPI)

Service users (patients) were very involved in the development of the HT&Me intervention and will continue to be involved throughout the RCT. Two of the co-applicant team are PPI representatives. The PPI co-applicants sit on the Trial Management Group (TMG) and participate in trial management decisions. We also established a Patient Advisory Group (PAG) consisting of 11 women who have previously been diagnosed with breast cancer and been prescribed AET (co-chaired by the PPI co-applicants). The PAG co-developed the content of the intervention and helped to test usability [26]. They continue to advise on patient-facing materials, recruitment rates and any emerging challenges, will review findings, and contribute to dissemination. Changes made as a result of the PAGs involvement include: adding images to the Participant Information Sheet (PIS) to show a diverse range of women; revision of language in the intervention animation to ensure it was clear to a lay audience; suggesting the addition of patient quotes about the lived experiences of side effects in the web-app; and improving the readability and lay understanding of the questionnaires. Further to this, we have set up a Community of Interest, a group of 28 women who have previously been diagnosed with breast cancer and have been prescribed AET, who advise on an ad hoc basis, via email, on a range of topics; for example, providing real-life hints and tips to deal with side effects for inclusion in the HT&Me intervention.

### Methods: participants, interventions, and outcomes

#### Study setting {9}

Recruitment is taking place in up to 80 NHS hospitals across the UK (England, Wales, Scotland, and Northern Ireland). The trial is recruiting through Acute Hospital NHS Trusts. In the UK, NHS breast cancer treatment is delivered through Acute Hospital Trusts in both secondary and tertiary care; these include district general hospitals, regional centres of excellence, tertiary referral centres, and teaching hospitals [42]. A list of trial sites can be obtained from ISCRTN: ISRCTN24852890. Sites which already offer intensive support for women taking AET (e.g. a specific clinic) were considered ineligible for the study.

### Eligibility criteria {10}

Patients are eligible if they meet all of the following criteria: aged 18 +; female; diagnosis of ER + ve invasive breast cancer, stages 1–3 and treated with curative intent; completed surgery for breast cancer; within 14 weeks of first post-surgery oral AET prescription (tamoxifen or aromatase inhibitor); completed chemotherapy (if applicable); able to access the internet and have access to an email address; willing to use a support package with a web-based component. The following women are also eligible providing they fulfil the above criteria, women: undergoing, or planned to receive radiotherapy; receiving anti-human epidermal growth factor receptor 2 (anti-HER2) therapies or ovarian suppression drugs; receiving, or planned to receive an adjuvant CDK4/6i (e.g. abemaciclib); have received neoadjuvant-AET; who have had a previous primary breast cancer (as long as they did not have AET to treat that first cancer).

Exclusion criteria are as follows: male; evidence of metastases (i.e. stage 4 disease); AET for a previous breast cancer; cognitive impairment sufficient to preclude participation as judged by the clinical team; or unable to read and understand English.

### Who will take informed consent? {26a}

Discussions about trial participation will take place during an in-person consultation or remotely, where the potential participant is given the opportunity to ask questions. Potential participants are provided with a copy of the PIS, and informed consent (written if in person or verbally, with a witness, if remote) is received by the local Principal Investigator (PI) (or trained designee) (see additional file). Women can refuse to participate without giving a reason. A copy of the signed consent form is provided to the participant (either by post, email or in person). The participant’s general practitioner (GP) is also then informed of their participation in writing.

To reduce digital exclusion, technical equipment (and technical support) such as tablets and data bundles are available to borrow (from the central co-ordinating team) for participants who do not have the required access to a digital device or the internet data to take part in the trial.

### Additional consent provisions for collection and use of participant data and biological specimens {26b}

Not applicable—no biological specimens are collected.

## Interventions

### Explanation for the choice of comparators {6b}

Usual care (control):

Participants randomised to usual care alone continue to be followed up as per institutional guidelines and follow-up processes. As breast cancer follow-up varies across sites, usual care at each site will be documented.

### Intervention description {11a}

**Fig. 1:**
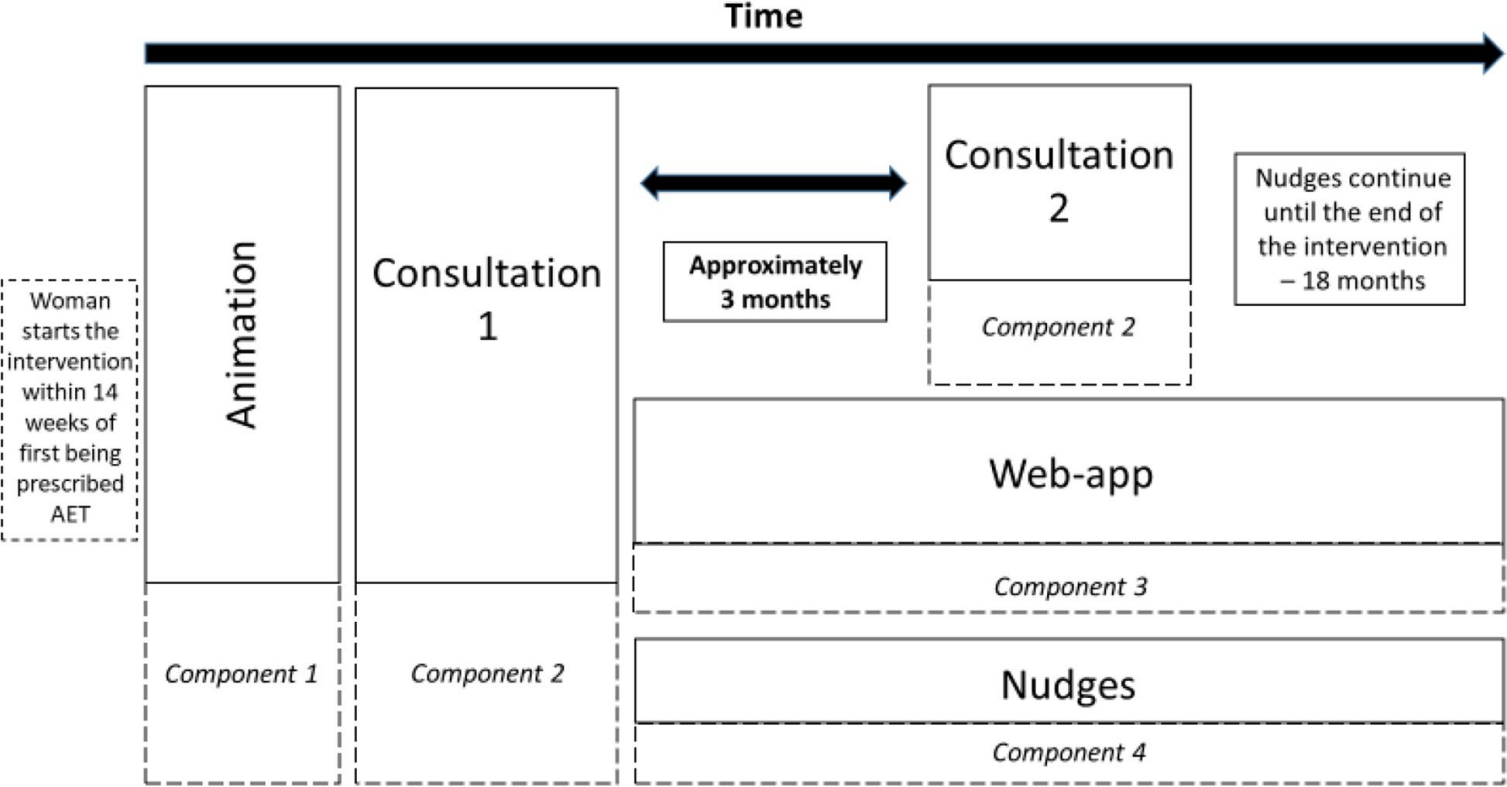
Overview of HT&Me intervention

HT&Me intervention:

The HT&Me intervention is summarised in Fig. 1.

#### Animation

Following randomisation, women in the HT&Me intervention are invited, via an email or letter, to view a 6 min animation which addresses necessity for AET and common AET concerns, and supports self-efficacy, motivation, goal-setting and developing an adherence habit. This is also housed on the HT&Me web-app and can be viewed as often as is desired by participants in the intervention arm.

#### Consultation 1

There are two models for the delivery of the consultations within the HT&Me intervention. In the ‘Site-Led Model’ participants randomised to the HT&Me intervention have an initial consultation with a ‘SWEET study practitioner’—who may be a breast care nurse, research nurse, or other health professional, based at the recruiting site, trained to deliver HT&Me. This consultation takes place face-to-face on site, unless the participant has a strong preference for a remote consultation. Where sites do not have the capacity or are unable to identify somebody locally to take on the role of SWEET study practitioner, they can opt for the ‘Breast Cancer Now (BCN) model’. BCN is a UK-based charity. Their services include a dedicated team of nurses who provide support online, on the phone or in person, to individuals affected by breast cancer [43]. For sites using the BCN model, the consultation is delivered remotely (via videoconference) by a BCN nurse who has been trained to deliver HT&Me. These initial consultations, which last for approximately 30 min, should take place within 4 weeks post-randomisation. The consultation date and time is confirmed by email or letter; this also include details on how to access the HT&Me animation (see above) so participants can watch this prior to their consultation. The consultation follow’s the consultation 1 guide (see additional file), and using elicitation, is tailored to the participants beliefs, concerns and AET-related behaviours. During Consultation 1, the participant is registered with a HT&Me account by the SWEET study practitioner/BCN Nurse and provided with unique log-in details. The SWEET study practitioner/BCN Nurse also introduces them to the web-app and provides support details in event of any IT problems. If they have not already accessed it, participants will view the animation at the consultation. Where requested, the participant is provided with a paper copy of the ‘how to’ guide for the web-app, which can also be found digitally within the HT&Me web-app. Following Consultation 1, the SWEET study practitioner/BCN Nurse complete a Consultation 1 appointment checklist (collating key information about the consultation, such as its duration). A sample of consultations are audio-recorded for the purpose of assessing fidelity.

#### HT&Me web-app

Intervention participants have access to the HT&Me web-app (hosted on a website) for the duration of their time in the trial. It contains tools and information to support adherence to AET. Features of the web-app include information on the importance of taking AET, supportive information on common side effects and coping strategies, how to talk to family members about breast cancer and AET as well as signposting to further support. It also contains interactive elements, such as ‘My Personal Support’ which asks questions to identify the participants current concerns and beliefs about AET and provides tailored support accordingly. Other interactive sections allow participants to set regular text or email reminders to take AET and order repeat prescriptions, as well as facilities to record AET side effects, noting their severity and impact. These can be used to support ongoing conversations with medical professionals, in addition to empowering participants to self-monitor their AET.

#### Consultation 2

Approximately 3 months after consultation 1, a follow-up consultation (approximately 15 min) with the SWEET study practitioner takes place by video call or telephone, or face-to-face if that is the participants preference. For sites using the BCN model, this consultation is performed remotely by a dedicated nurse at BCN. This follows the Consultation 2 guide (see additional file) and communicates the continuing importance of treatment and addresses any emerging AET-related concerns or issues. The consultation is tailored around the participant’s current necessity and concern beliefs about, and experiences of, AET. Following the consultation, the Consultation 2 checklist is completed by the SWEET study practitioner/BCN Nurse. A sample of consultations are audio-recorded for the purpose of assessing fidelity.

Participants may be asked to provide brief feedback using an automated SMS service, rating the usefulness of these appointments using a numerical scale. This will provide feedback on the consultation which may not easily be recalled at the point of the follow-up questionnaire (see Participant Timeline section).

#### Nudges (Motivational messages)

At regular intervals, participants are sent an email or text message (according to participant preference) which provides tailored prompts for adherence, reinforces the importance of continuing AET, and indicates support is available if needed via the web-app. These are sent at 2 weeks and 1-month post-consultation 1, and then once per month for the 18 month duration of the trial.

The core components of the intervention, their mode and frequency of delivery and theoretical underpinning have been summarised in Table 2 (additional file). It should be noted that PaPA provides a theoretical underpinning for the whole intervention; the individual behaviour change techniques, which relate to separate components and specific aspects of the HT&Me web-app have been categorised using the Behaviour Change Taxonomy [44]. Further details are published elsewhere [26].

**Fig. 2:**
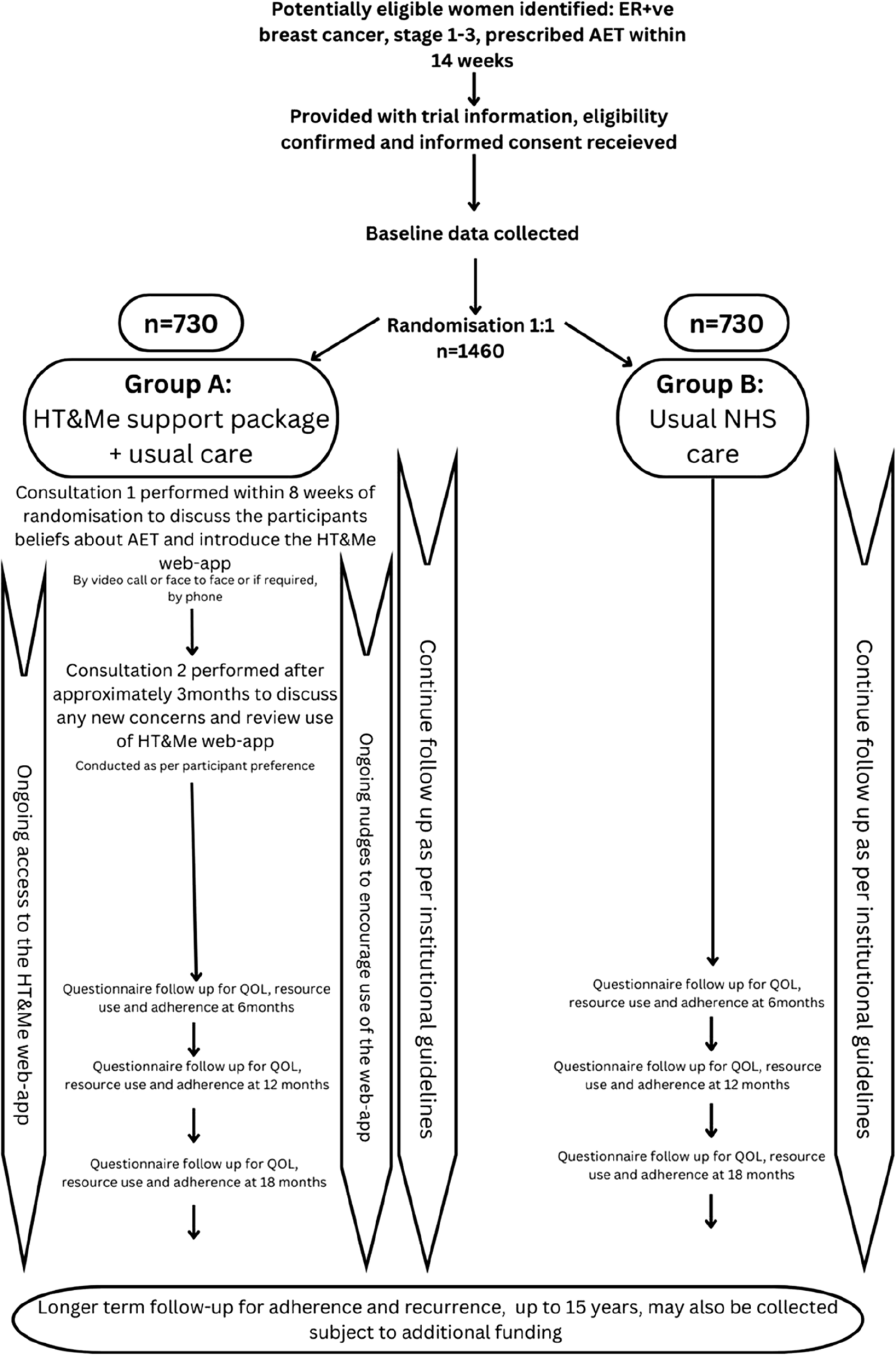
The participant pathway

The HT&Me logic model and mechanisms of action through which we expect it to improve adherence and HRQoL, and ultimately to reduce breast cancer recurrence and NHS costs, is shown in Fig. 2 (Additional file).

### Criteria for discontinuing or modifying allocated interventions {11b}

We do not anticipate any scenario whereby participation will be discontinued or have intervention allocation modified by the trial team.

Participants may withdraw from participating in the trial at any time, at their request, without prejudice. Unless a participant also explicitly withdraws their consent for data linkage (see Outcomes section), they are followed-up as planned.

### Strategies to improve adherence to intervention {11c}

To optimise fidelity, the SWEET study practitioners/BCN Nurses are trained in delivering the HT&Me intervention; training is hosted on the NHS Learning Hub. SWEET study practitioners/BCN Nurses attend a one-hour online training overview, followed by 7–8 h of independent online learning, which includes time to become familiar with the web-app. A paper training manual supplements the online information. SWEET study practitioners/BCN Nurses can log training hours for revalidation or equivalent, and for continued professional development. One aspect of this training includes the building of rapport with participants during Consultation 1. As part of this sites and BCN are asked, where possible, to ensure continuity in personnel between consultations 1 and 2. This relationship between SWEET study practitioner/BCN Nurse and participant is expected to support adherence to the intervention.

In recognition of the importance of tailoring in supported self-management interventions, HT&Me has been designed to incorporate tailoring as an important element of the intervention. For example, the Consultations are tailored to the individual participant’s beliefs and concerns about AET; they are also shown the My Personal Support section within the web-app, during Consultation 1, which helps participants find the elements of the web-app that are most useful for them at that time.

The regular nudges (motivational messages) delivered via email or text message within the intervention are also anticipated to support adherence to the intervention.

To promote adherence to the consultation aspect of the intervention, participants are sent an appointment confirmation letter for both Consultation 1 and 2. If the date and time no longer suit, there is the opportunity to reschedule. If a participant does not attend a consultation, a further appointment date can be arranged, with the option to reschedule again if unsuitable.

### Relevant concomitant care permitted or prohibited during the trial {11d}

There are no limits on the concomitant care that patients can receive during the RCT, as long as the participant meets the inclusion criteria. Usual care continues for both trial arms.

### Provisions for post-trial care {30}

Participants in the intervention arm have access to the web-app component of the intervention for the 18 months they are within the trial.

As we hope to secure funding for longer term follow-up (e.g. to examine effects of the intervention on adherence at 5 years), we decided not to use a wait list control design (i.e. one which permits access to the intervention for usual care participants after the trial is completed).

### Outcomes {12}

Two primary outcomes are assessed: (i) poor AET adherence and (ii) cancer-specific HRQoL. Adherence is measured using a combination of an objective measure (proportion of days covered (PDC) based on prescription encashment data^1^ routinely recorded by the NHS from community pharmacy records) and a subjective measure (self-report using MARS-5 [31]) over 18 months post-randomisation. Combining objective and subjective measures of adherence is considered the gold standard approach and allows misclassification to be reduced [45]. Participants will be classified, for analysis, as showing some degree of non-adherence (henceforth ‘inadequately adherent’; (PDC < 80%) OR (PDC ≥ 80% and a total MARS-5 score of ≤ 23)) or ‘adequately adherent’ (PDC ≥ 80% AND a total MARS-5 score of > 23) [46]. Cancer-specific HRQoL will be assessed using the FACT-G questionnaire [32]. Primary and secondary outcomes and potential mediators are specified in Table 1.

#### Health economic evaluation

Women complete the EQ-5D-5L [34] and report the resource use that they consider to be related to their breast cancer or side effects/complications at baseline, 6, 12, and 18 months. The resource use questionnaire was adapted from the UK Cancer Costs Questionnaire [47] and pre-tested among patients, clinicians and other members of the project team. AET costs will be based on prescription data. A within trial cost-utility analysis will estimate the cost per quality-adjusted life-year (QALY) gained. For HT&Me vs. usual care the base case analysis will take an NHS and personal and social services perspective; a sensitivity analysis will explore a societal perspective. EQ-5D-5L-related utilities, resource use and costs to the NHS, patients and society will be reported. Resource use and costs will be scaled-up to ascertain a national NHS cost/budget impact.

#### Process evaluation

Using a mixture of qualitative and quantitative methods, the process evaluation will explore fidelity of the intervention as delivered, received, and enacted; assess whether the intervention worked as hypothesized by the logic model; and identify any moderating contextual factors and/or unintended consequences of the intervention.

To maximise our understanding of usual care and contextual factors, sites are asked to complete a short questionnaire when they first join the study and at the end of recruitment to describe current practices in relation to any support routinely provided to women regarding endocrine therapy. As the trial is pragmatic and set against ‘real world’ practice, we do not plan to include this data in the statistical analysis related to the primary outcomes. Instead, this data will be considered descriptively within the mixed method process evaluation as it captures contextual issues that may influence, for example, the types of hospitals/services or patients for whom the intervention ‘works’.

To understand mechanisms of action of the intervention, structural equation modelling approaches will be used to estimate the proportion of the intervention effect on the primary outcomes that occurs via the putative mediator variables that are the target of the intervention (Table 1). This analysis will inform understanding of whether, and to what extent, the mediator variables were influenced by the intervention and, hence, how the intervention ‘worked’.

Semi-structured interviews will also be conducted during the trial with participants (intervention arm, *n* = 25–30; usual care arm, *n* = 25–30) and SWEET study practitioners and other key health professionals (*n* = 25–30). These will explore (as appropriate) the following: views and experiences of the intervention and the trial overall, as well as contextual factors. Furthermore, these interviews will explore what would need to be in place (i) to maintain the intervention as a part of routine care in the absence of the research team; and (ii) to provide the intervention to participants who do not speak English. Interviews will be analysed thematically using the Framework Approach [48].

Further to this, within the follow-up questionnaires (see Participant Timeline section), participants are asked for their opinions on how helpful they found the different components of the intervention.

Finally, bespoke anonymised web-app analytics are recorded to determine how often individual participants use the web-app and which elements or pages they access.

#### Longer term follow-up

Participants provide consent to be followed up for up to 15 years. They will be flagged through NHS databases, in the four nations, to identify hospital admissions (e.g. via Hospital Episode Statistics (HES) in England) and deaths (e.g. via Office of National Statistics mortality records in England). Separate funding will be sought for longer-term follow-up to assess adherence and breast cancer deaths, and survival, beyond the end of the current programme of research.

### Participant timeline {13}

The participant pathway can be seen in Fig. 2.

After providing consent, participants are asked to complete the baseline questionnaire, consisting of self-reported measures described in Table 1. This can be completed electronically, or in a paper format. Once complete, participants are randomised as described in the Sequence Generation section below. Those in group A participate in the HT&Me intervention, as described above. All participants are asked to complete a follow-up questionnaire at 6, 12, and 18 months, which repeat the questions from the baseline (follow-up questionnaires can be completed electronically, or in a paper format). A representative sample of participants from both groups will be invited to participate in process evaluation interviews as described above. Long-term follow-up (for up to 15 years) will be conducted via data linkage (dependent on additional funding). The Schedule of Assessments can be seen in Table 3 (Additional file).

### Sample size {14}

The trial is powered to detect differences in both of the primary outcomes (AET adherence and cancer-specific HRQoL). Assuming 50% of eligible patients consent, we anticipate approaching 2920 potential participants to provide a recruitment target of 1460, comprising 730 randomised to each arm. With an estimated 15% loss to follow-up in each arm, the primary outcomes will be measured for 620 participants per arm. With *α* = 0.05 (two-sided test), 620 participants in each arm provides 90% power to detect as statistically significant a difference of 5% in inadequate adherence (e.g. consistent with a reduction from 10% in the usual care arm to 5%). A change of 3–7 points on the FACT-G, the primary outcome measure for cancer-specific HRQoL, is considered clinically significant [32], and 620 participants in each arm will provide 91% power to detect a difference of 3 points in FACT-G scores between arms (*α* = 0.05, two-sided test; assuming mean FACT-G score is 83.9 (SD = 15.9) in the usual care arm (personal communication—author CC).

### Recruitment {15}

Potentially eligible participants are either identified via multi-disciplinary team meetings, via hospital records or in hospital clinics. A member of the local team at each site reviews the potential participants’ medical records to assess eligibility. As previously described, a PIS is provided by the local research team, either in person or by post or email, and informed consent gained. All women considered potentially eligible and approached are added to site screening logs.

## Assignment of interventions: allocation

### Sequence generation {16a}

Local research teams at sites randomise each participant on the online automated SWEET randomisation portal, hosted by the SWEET trial office. Participants are randomised on a 1:1 basis to the HT&Me intervention plus usual care, or to usual care alone. The statistician checks the allocation intermittently to ensure randomisation is balanced across the trial arms. Trial arms are allocated randomly using a bespoke computerised minimisation algorithm held centrally at the SWEET trial office and stratified, in line with previous research [11, 17, 25] and recommendations from our Clinical Reference Group, as follows:

1. Age: < 50, 50 +
2. AET type: tamoxifen/AI
3. Treatment complexity

i. No chemotherapy, no anti-HER2, no CDK4/6i.
ii. Chemotherapy, no anti-HER2, CDK4/6i.
iii. Chemotherapy, anti-HER2, no CDK4/6i.
iv. Chemotherapy, no anti-HER2, no CDK4/6i.
v. No chemotherapy, no anti-HER2, CDK4/6i.

### Concealment mechanism {16b}

Randomisation takes place via a secure automated link—thus the mechanisms of generating the allocation sequence and randomisation patterns are concealed from those doing the randomisation at the NHS site, as well as all other research team members.

### Implementation {16c}

After randomisation has been undertaken, the local research team inform the participant if they have been randomised to the intervention plus usual care arm, or the usual care alone arm.

## Assignment of interventions: blinding

### Who will be blinded {17a}

Not applicable—this is an unblinded trial. Our primary (and main secondary) outcomes are either patient reported (e.g. HRQoL/self-reported adherence) or objectively measured using health service data (e.g. PDC). Following a patient centred approach, for trial outcomes which were not assessed using objective health service data, we determined that these should be assessed by the patients themselves, rather than (for example) their clinicians. It was our intention to create an ‘environment’ where women would feel able to honestly report how they were feeling, and whether they had been taking their AET; therefore, we used self-completed PROMs rather than questionnaires administered by someone in the hospital or research team. As women knew which arm they were in, this was therefore unblinded.

### Procedure for unblinding if needed {17b}

Not applicable—this is an unblinded trial.

## Data collection and management

### Plans for assessment and collection of outcomes {18a}

Site staff are expected to collect key clinical information (including information about diagnosis, tumour histology, surgery, relevant planned adjuvant treatment) and demographic information at baseline using approved case report forms. Self-reported outcomes are collected from participants via questionnaire at baseline, 6, 12, and 18 months, as described above. All tools and scales within the questionnaires were previously validated or have been adapted from validated instruments to fit this specific context and patient group.

### Plans to promote participant retention and complete follow-up {18b}

All trial follow-up data can be collected remotely, to minimise participant burden and promote participant retention. Up to two reminders (by phone or email) to complete the follow-up questionnaires may be sent if needed. Where participants are deemed as lost to follow-up, data will continue to be collected remotely where possible using hospital records unless a participant explicitly withdraws their consent.

A dedicated email address will be provided to participants for support with any technical difficulties relating to the HT&Me web-app. All research contacts will aim to build a positive relationship and experience for participants.

### Data management {19}

SWEET uses electronic remote data capture (via the bespoke SWEET database). Data entered by site staff is reviewed by the SWEET trial office, with actions outlined in the data management plan to protect participant safety, compliance and the integrity of the data. In-built validations, agreed with the co-CIs and trial statistician, have been used within the database to reduce the risk of ineligible participants being recruited and illogical or incorrect data being entered. The trial manager (or delegate) will regularly produce reports to monitor data completeness and quality, and missing data or discrepancies will be raised with the relevant site.

Questionnaires can be completed by the participant directly either using an electronic questionnaire which populates into the trial database, or on paper if they prefer. All paper forms are returned to the SWEET trial office where they are entered onto the secure database by a member of the trial team—primarily the data entry clerk. The trial team will conduct ad hoc spot checks to double check entry. The paper forms are securely stored in order of trial number in locked filling cabinets in the secure trial office and are transferred to the secure archiving room once entered. The online system can be accessed by the trial team via individual login with access rights assigned accordingly. Ongoing data entry quality control checks are made at agreed intervals. All documents will be stored safely in confidential conditions as outlined below.

### Confidentiality {27}

The trial is conducted in accordance with the current UK General Data Protection Regulation, and participant anonymity is maintained throughout, and following completion of the trial. Participants are allocated a study specific trial number which is used to identify them on all trial documents and on the database. A full back up of the database is run every day, in additional to transactional backups every 15 min.

Participants consent to provide their name, NHS number (or devolved Nation equivalent), date of birth, GP and personal contact details (telephone number and email address). Access to this data is restricted to trial staff and authorised personnel only, this includes the central research team who are conducting the trial, overseeing HT&Me accounts and performing NHS (or other devolved Nation equivalent) data linkage, the BCN team (where BCN are responsible for conducting the SWEET consultations), and the research team responsible for conducting the process evaluation interviews. Trial data, including personal identifiable information, is securely stored on the SWEET database, developed and maintained by the Warwick Clinical Trials Unit Programming Team.

The HT&Me web-app is securely hosted online on Microsoft Azure (housed in the UK) with personal data stored within a two-layer encrypted folder. The information collected on use of the web-app (analytics) will be anonymous; IP addresses are not collected. Linked datasets obtained from NHS England (or devolved Nation equivalents) are pseudonymised and securely stored to enable analysis.

Process evaluation interviews will be audio-recorded. These, and any recordings of SWEET consultations, will be securely transferred from the recording device to a secure laptop and uploaded to the trial area on the secure server of the SWEET trial office. Recordings will then be deleted from their original source.

### Plans for collection, laboratory evaluation, and storage of biological specimens for genetic or molecular analysis in this trial/future use {33}

Not applicable—biological specimens are not collected.

## Statistical methods

### Statistical methods for primary and secondary outcomes {20a}

The data will be analysed in line with the statistical and health economics analysis plan, which will be reviewed and approved by the Data Management Committee (DMC) prior to implementation. Primary analysis will be undertaken on an intention-to-treat basis. Characteristics of participants will be examined by arm. The analysis of the primary endpoints of AET adherence and cancer-specific HRQoL at 18 months will be analysed using logistic and linear regression methods respectively, adjusted for the stratification variables of age, AET type and treatment complexity, as categorised by the computer minimisation algorithm. The size of any intervention effect on each primary outcome over time will be estimated using mixed-effects logistic and linear regression methods (as appropriate), adjusted for stratification variables. Underlying assumptions of the regression models will be investigated, reported and addressed. Different measures of adherence will be considered in sensitivity analyses (e.g. extent of adherence, treating PDC/MARS-5 as continuous variable; early discontinuation, both self-reported and using encashment records; Table 1). Using the encashment data, we will create a longitudinal daily history of AET availability for each trial participant by assigning the days’ supply in each encashed prescription to sequential days from date of randomisation to end of follow-up. This approach takes into account the length of any gaps in medication availability. The full and detailed calculation of this will be detailed within the trial’s Statistical Analysis Plan. A two-sided 5% level of statistical significance will be used throughout.

### Interim analyses {21b}

Not applicable—no interim analysis is planned for this trial.

### Methods for additional analyses (e.g. subgroup analyses) {20b}

Pre-specified sub-group analyses defined by the previously described stratification variables will be undertaken using appropriate modelling techniques. These exploratory sub-group analyses will have lower power than the main whole trial analysis; they will be considered hypothesis-generating and results scrutinised graphically via forest plots.

### Methods in analysis to handle protocol non-adherence and any statistical methods to handle missing data {20c}

Due to the potential biases associated with complete case analyses, consideration will be given to the amount of missing data within the trial. A sensitivity analysis will determine the degree to which the conclusions may change with different missing data assumptions and mechanism models and consideration given to appropriate multiple imputation if necessary. Non-adherence to AET is a primary endpoint of the trial and analysis of this is covered in the ‘Statistical methods for primary and secondary outcomes’ section.

### Plans to give access to the full protocol, participant level-data, and statistical code {31c}

A public website has been developed to promote and disseminate the programme of research and its findings https://www.sweetstudy.co.uk/. This also contains the key trial documents (e.g. PIS, privacy notice). The Health Research Authority (HRA) approved full trial protocol is available from the ISRCTN website (ISRCTN-ISRCTN24852890: Supporting women with adherence to hormone therapy following breast cancer).

Anonymised quantitative datasets and statistical code generated during the trial will be made available through an NIHR recommended open access platform.

## Oversight and monitoring

### Composition of the coordinating centre and trial steering committee {5d}

The TMG includes those individuals responsible for the day-to-day management of the trial, including the co-CIs, statistician, health economic lead, trial manager, trial co-ordinator, and data manager. The TMG meet on a monthly basis to monitor all aspects of trial conduct and progress, ensure that the protocol is adhered to and take appropriate action when necessary to ensure the quality of the trial.

A Trial Steering Committee (TSC) has been established to provide overall oversight for the trial on behalf of the Sponsor and Funder and ensure that the project is conducted to the rigorous standards set out in the Department of Health’s Research Governance Framework for Health and Social Care, and the Guidelines for Good Clinical Practice. It includes four independent members (Chair (who is a breast cancer clinician), Statistician, PPI representative and Other Independent Member), members of the research team, and representatives of Sponsor and Funder.

The full remit and responsibilities of the TSC is documented in the Committee Charter and has been signed by all members. The independent members previously comprised the Programme Steering Committee which was established when the programme of research commenced (2020), so they are familiar with the phases of the programme, including the RCT. As required by Funder, the TSC will meet 6 monthly; additional ad hoc meetings may take place if required.

### Composition of the data monitoring committee, its role and reporting structure {21a}

A Data Monitoring Committee (DMC) has been established, comprising three independent members (Chair (who is a breast cancer clinician), Statistician and other independent academic) plus trial representatives as required. The DMC will meet to review trial progress, recruitment, protocol compliance and interim assessment of outcomes, annually or more frequently if requested. The DMC will advise the TSC whether the trial should continue, be amended or stop prematurely based on the monitoring of trial data and any future publications or emerging worldwide evidence. The full remit and responsibilities of the DMC, and any conflicts of interest, are documented in the Committee Charter which has been signed by all members. A final DMC meeting will be held upon the availability of the final trial data, following the final data lock, and cleaned datasets being made available to trial statisticians.

### Adverse event reporting and harms {22}

All treatments administered to participants are identical to the treatment given in usual clinical practice, for which there is extensive safety data already available. Due to the low-risk nature of the intervention, adverse event reporting will not be undertaken.

### Frequency and plans for auditing trial conduct {23}

A Trial Monitoring Plan has been developed and agreed by the TMG based on the trial risk assessment. Monitoring activity will be predominantly undertaken centrally (e.g. review of screening logs, recruitment data and case report forms) with remote visits to sites scheduled if required. A representative from the SWEET trial office will work with the site staff to resolve issues, offer appropriate training if necessary, and determine the site’s future participation in the trial.

All forms are checked for completeness and consistency and any anomalies are queried with the site. The trial staff maintain regular communication with sites through routine calls, mailings, and monthly meetings with sites, focussing on different topics, sharing good practice and successful strategies.

### Plans for communicating important protocol amendments to relevant parties (e.g. trial participants, ethical committees) {25}

All amendments are documented by the SWEET trial office. After Sponsor approval, substantial amendments are submitted for HRA Approval, which includes NHS Research Ethics Committee review, prior to communication to relevant participating sites. Amendments deemed non-substantial by Sponsor are submitted to the HRA, and the applicable national coordinating functions in the devolved administrations, for review. Each trial site must ensure that they are using the most up to date version of the protocol, the PIS and informed consent forms.

Where the introduction of protocol amendments and the availability of important new information may be relevant to the participant’s willingness to continue taking part in the trial, participants will be made aware of these amended documents, which will be updated on the SWEET public website.

### Dissemination plans {31a}

Our dissemination strategy includes conference presentations, scientific papers, lay communications, and briefings (paper, digital and events) for clinical teams, patient groups, and health service decision-makers. Our public-facing SWEET website contains information for both scientific and lay audiences.

For scientific dissemination, the research findings will be presented at relevant national and international meetings and papers will be submitted to open access journals. Papers and conference presentations will be publicised on the public website.

For lay dissemination, participants will be given the option of receiving a lay summary of the findings once the trial is completed. To reach patient and general populations, updates will be posted on the public website, with key messages (crafted together with the PPI group; see Public and Patient Involvement section) highlighted. We plan to hold a dissemination event for breast cancer survivors, healthcare professionals, and relevant health service decision-makers. If there is sufficient interest, we will live stream this event to other locations (e.g. collaborating centres). We may also record parts of the event and post on the website.

## Discussion

The SWEET RCT has been preceded by intervention optimisation studies [26] and a feasibility study [27], which helped to inform the development of the RCT to ensure the deliverability of the trial. There have been a few challenges in the development and delivery of the trial thus far; these are discussed below.

To address the issue of some sites wishing to participate, but not having the capacity to deliver the intervention, in partnership with BCN (Breast Cancer Now—UK Charity), we developed a centralised model of intervention delivery, whereby the intervention is delivered online by BCN nurses who have been trained by the trial team. This option worked well in the feasibility study, and this model is being utilised by a large number of sites in the RCT. The BCN model also helps reduce the risk of contamination between study arms in sites, although sites are asked wherever possible, that the designated SWEET study practitioner does not generally support women with AET as part of their usual role.

We are also cognisant of the variation in usual care around the UK, and that usual care is evolving as our trial progresses. Increasingly, sites are recognising that women require greater support with managing AET and some are now putting in place in-house interventions that offer support. This has the potential to dilute the effect of the intervention. Our trial is pragmatic and takes place within the context of real-world practice so, following the advice of our TSC, unless sites have put in place intensive support for women taking ET, they will be eligible to participate.

Establishing the best way to measure AET adherence is not straightforward. There are no simple, and feasible, biomarkers and following a review of the best alternative options we opted for a composite measure bringing together encashment data (shown to be a reasonable surrogate for utilisation) with self-report to reduce misclassification. However, there are recognised challenges with obtaining routine NHS data, at least in England and the data sources across the four nations vary. Moreover, despite strategies in place to maximise questionnaire completion rates, not all participants will complete questionnaires, so self-report will be missing for some. The Statistical Analysis Plan will account for this.

A potential limitation of the trial is the requirement for access to an email address, willingness, and ability to access the internet (although a device with data and technical support can be provided) and ability to read and understand English in order to take part. These issues were discussed at length when designing the trial, but were ultimately considered necessary inclusion criteria in order for the trial to be feasible to conduct within the available budget. However, as many steps as possible have been taken to minimise digital exclusion; for example, in developing HT&Me, we placed considerable focus on ensuring navigation around the webapp [26] was intuitive and user-friendly.

If, as we hope, the intervention is shown to be effective and is implemented at scale in the future, access to an email address will not be a requirement. We will also explore ways to make the content of the webapp available in other formats for those who cannot or do not wish to access the internet. We also plan to seek additional funding to translate and, if required, culturally adapt, the intervention for people who are not able to read or understand English.

The past two decades have seen an explosion in availability of oral treatments for cancer, including a wide range of biological and precision therapies. Evidence is accumulating that adherence to some of these drugs may be suboptimal, at least in some patient subgroups [49–52]. The approaches used in SWEET—from intervention development to measuring adherence—and the trial findings, have the potential to inform how best to deliver adherence support for these other anti-cancer therapies.

Trial recruitment commenced in spring 2024, and recruitment is going well, with minimal issues being raised by sites. Informal feedback from the local research teams is very positive.

In conclusion, this trial has potential to improve quality of life in women with breast cancer and, by improving adherence to endocrine therapy, to reduce numbers of recurrences and breast cancer deaths, benefiting women, their families and the health service.

## Trial status

Protocol Version 4.0 08.04.2024, recruitment began on 28.03.2024, anticipated end date for recruitment is Autumn 2025. At time of submission for publication we were open in 66 sites and had recruited 1342 women. Current recruitment figures are available on the public website: https://www.sweetstudy.co.uk/.

## Supplementary Information

Additional file 1: Table 2. Intervention components, mode and frequency of delivery, mechanisms of action and behaviour change techniques.

Additional file 2: Figure 2. Logic Model.

Additional file 3. Table 3–Schedule of Assessments.

Additional file 4. Consultation 1 (Initial appointment) with SWEET study nurse: Nurse’s Guide.

Additional file 5. Consultation 2 (follow-up appointment) with SWEET study nurse: Nurse’s Guide.

Additional file 6. Template consent form.

## Data Availability

Quantitative datasets generated during the trial will be made available through an NIHR recommended open access platform. The protocol and other trial materials are available on the public website https://www.sweetstudy.co.uk/. The full protocol is available via ISRCTN.

## Abbreviations

AET: Adjuvant endocrine therapy
BCPT: Breast Cancer Trialist Prevention checklist
BMQ-AET: Beliefs about Medicines Questionnaire-Adjuvant Endocrine Therapy
BCN: Breast Cancer Now
CS-SES: Cancer Survivor Self-Efficacy Scale
DMC: Data Monitoring Committee
ER: Oestrogen receptor
FACT-G: Functional Assessment of Cancer Therapy Scale-General
GP: General Practitioner
HRA: Health Research Authority
HRQoL: Health-Related Quality-of-Life
HES: Hospital Episode Statistics
IPQ-BCS: Illness Perception Questionnaire for Breast Cancer Scale
ISRCTN: International Standard Randomised Controlled Trial Number
MARS-5: Medication Adherence Report Scale
NHS: National Health Service
NICE: National Institute for Health and Care Excellence
NIHR: National Institute for Health and Care Research
PaPA: Perceptions and Practicalities Approach
PDC: Proportion of Days Covered
PI: Principal Investigator
PIS: Patient information sheet
PMC: Programme Management Committee
PPI: Patient and public involvement
QALY: Quality-Adjusted Life Year
QoL: Quality of Life
RCT: Randomised Control Trial
SIMS: Satisfaction with Information about Medicines Scale
TMG: Trial Management Group
TSC: Trial Steering Committee

## Footnotes

1 Medication encashment datasets comprise records of prescribed medicines which are dispensed by a pharmacist and collected by the patient.
