# Supplementary material for: Improving outcomeS for Women diagnosed with early breast cancer through adhErence to adjuvant Endocrine Therapy (SWEET): study protocol for a pragmatic randomised control trial of a patient-centred intervention to improve adherence to endocrine therapy in early breast cancer
Source: Trials. 2025 Nov 26;26:551. doi: 10.1186/s13063-025-09056-6 (PMC12659038; doi:10.1186/s13063-025-09056-6)
Supplement: Supplementary file 1 — Additional file 1: Table 2. Intervention components, mode and frequency of delivery, mechanisms of action and behaviour change techniques. [file 13063_2025_9056_MOESM1_ESM.docx]

Table 2 Intervention components, mode and frequency of delivery, mechanisms of action and behaviour change technique

| **Intervention component** | **Mode of delivery** | **Frequency of delivery** | **Mechanism of action examples^2^** | **Behaviour change technique examples^1,2^** |
| --- | --- | --- | --- | --- |
| Animation:  - 6 minute video explaining what AET is, how it works, and the importance of taking it every day | - Invited to view online via email or letter  - Additionally housed on web-app | - Invited to view following randomisation, and during consultation 1 - Viewed anytime via web-app | - Increase knowledge about AET  - Increase necessity beliefs  - Reduce concerns  - Remove practical barriers to taking AET | - Action Planning  - Social Support (unspecified)  - Information about Health Consequences  - Habit formation |
| Consultation 1:  - Approximately 30 minutes  - Tailored to the participants beliefs, concerns and AET-related behaviours  - Participants registered on HT&Me, given unique log-in and introduced to web-app  - Invited to view animation | Site-Led Model:  - face to face consultation with SWEET study practitioner  Breast Cancer Now (BCN) model:  – remote video consultation with BCN study nurse | - Once - within 4 weeks post-randomisation | - Increase knowledge about AET  - Increase necessity beliefs  - Reduce concerns  - Address beliefs about consequences of not taking AET  - Empower women to know where they can seek support | - Information about health consequences  - Habit formation  - Verbal persuasion about capability  - Social support (unspecified)  - Social support (practical) |
| HT&Me web-app:  - Contains tools and information to support AET adherence  - Includes information on the importance of taking AET, side effect support  and coping strategies, techniques to discuss AET with others, as well as signposting to further support  - Interactive elements include ‘My Personal Support’ which identifies concerns and beliefs about AET and provides tailored support accordingly, setting up text or email reminders to take AET or order repeat prescriptions and facilities to record AET side effects | Online - accessible on mobile phone, tablet, desktop or laptop computer | Intervention participants have access to the HT&Me web-app for the duration of their time in the trial. | - Increase necessity beliefs  - Address beliefs about consequences of not taking AET  - Behavioural regulation (e.g. habit formation)  - Increase self-efficacy for taking AET  - Provide coping strategies for side-effects  - Increase confidence in managing side-effects  - Improve QoL  - Improve physical activity/diet  - Reduce emotional distress associated with cancer  - Improve social support  - Improve relationship with HCPs  - Reduce forgetting | - Goal setting  - Problem solving  - Review behaviour goal(s)  - Self-monitoring of behaviour  - Self-monitoring of outcome(s) of behaviour  - Information about antecedents  - Re-attribution  - Behavioural experiments  - Prompts/cues  - Credible source  - Reduce negative emotions  - Restructuring the physical environment  - Distraction  - Framing/reframing  - Focus on past success  - Self-talk |
| Consultation 2:  - Approximately 15 minutes  - Communicates the continuing importance of treatment  -Addresses any emerging AET-related concerns or issues  - Tailored around the participant’s current necessity and concern beliefs about AET | Site led model:  - Video call or telephone, or face-to-face (according to participant preference).  BCN model:  - Video call or telephone, performed remotely by BCN nurse | Once - approximately three months after consultation 1 | - Increase knowledge about AET  - Increase necessity beliefs  - Reduce concerns  - Address beliefs about consequences of not taking AET  - Empower women to know where they can seek support | - Information about health consequences  - Habit formation  - Verbal persuasion about capability  - Social support (unspecified)  - Social support (practical) |
| Nudges:  -Motivational messages  -Reinforces the importance of continuing AET  -Indicates support is available via the web-app | Email or text message (according to participant preference) | Two weeks and one-month after consultation 1, then once per month for the 18 months | - Increase knowledge about AET  - Increase necessity beliefs  - Reduce concerns  - Remove practical barriers to taking AET | - Action planning  - Prompts/cues  - Habit formation  - Problem solving |

^1^Behaviour change techniques categorised using the Behaviour Change Taxonomy^44^

^2^Further detail is reported elsewhere^26^
