# Supplementary figures and images for: Improving outcomeS for Women diagnosed with early breast cancer through adhErence to adjuvant Endocrine Therapy (SWEET): study protocol for a pragmatic randomised control trial of a patient-centred intervention to improve adherence to endocrine therapy in early breast cancer
Source: Trials. 2025 Nov 26;26:551. doi: 10.1186/s13063-025-09056-6 (PMC12659038; doi:10.1186/s13063-025-09056-6)

Figure 2 Logic Model

*
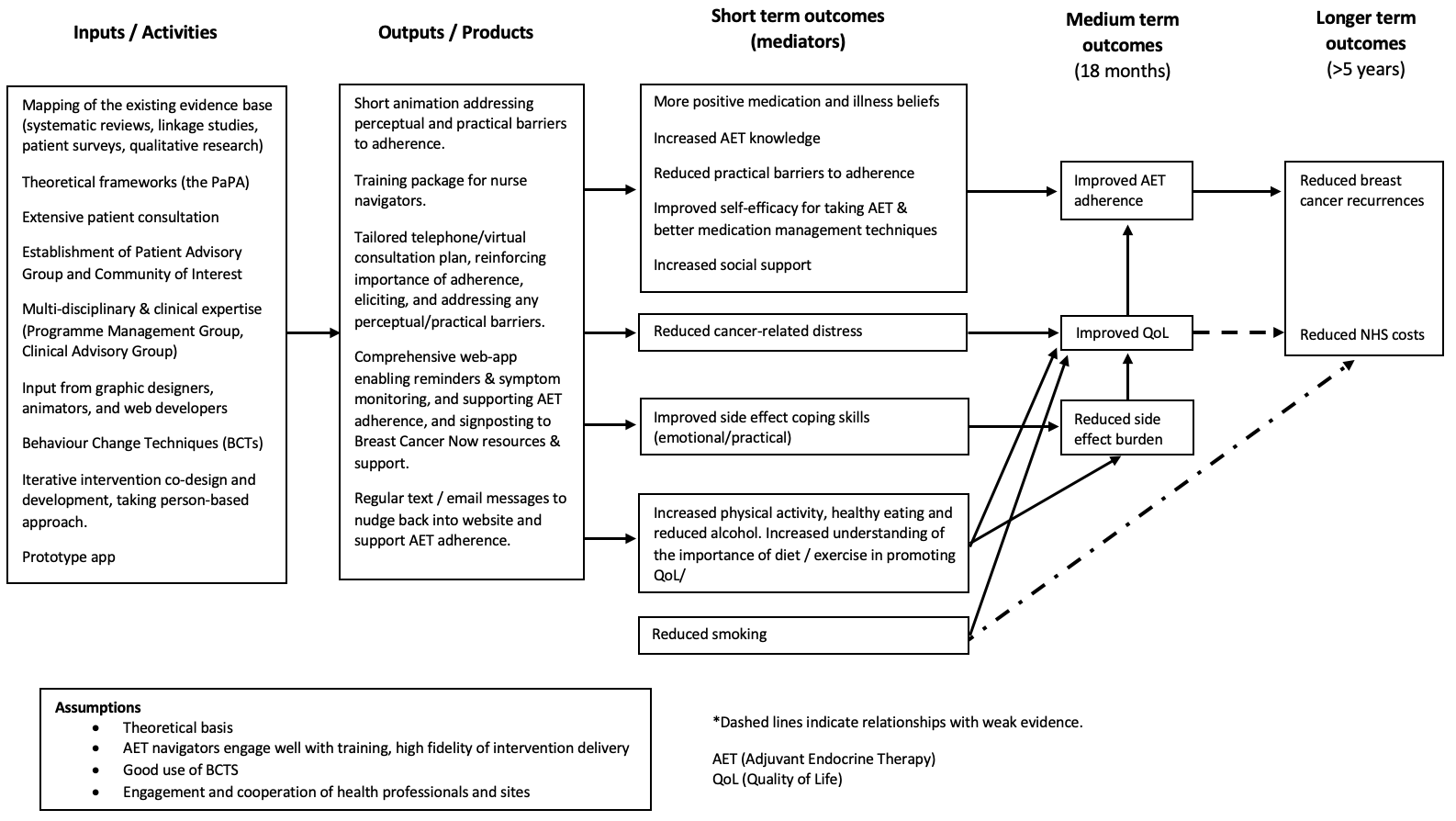
*

Supplement: Supplementary file 2 — Additional file 2: Figure 2. Logic Model. [file 13063_2025_9056_MOESM2_ESM.docx]
