# Supplementary material for: Improving outcomeS for Women diagnosed with early breast cancer through adhErence to adjuvant Endocrine Therapy (SWEET): study protocol for a pragmatic randomised control trial of a patient-centred intervention to improve adherence to endocrine therapy in early breast cancer
Source: Trials. 2025 Nov 26;26:551. doi: 10.1186/s13063-025-09056-6 (PMC12659038; doi:10.1186/s13063-025-09056-6)
Supplement: Supplementary file 3 — Additional file 3. Table 3–Schedule of Assessments. [file 13063_2025_9056_MOESM3_ESM.docx]

Table 3 – Schedule of Assessments

| Visit | 1 | 2 | 3 | 5 | 6 | 7 | 8 |
| --- | --- | --- | --- | --- | --- | --- | --- |
| Visit Window  (No. Weeks ± No. Days) | Baseline | Week 4  (+4 weeks) | Week 16  (+4 weeks) | 6 Months  (+/- 1 month) | 12 Months  (+/- 1 month) | 18 Months  (+/- 1 month) | Long-term follow-up  *Annually up to 15years** |
| Informed consent | x |  |  |  |  |  |  |
| Medical history | x |  |  |  |  |  |  |
| Inclusion/exclusion criteria | x |  |  |  |  |  |  |
| Questionnaire booklet | x |  |  | x | x | x |  |
| Randomisation | x |  |  |  |  |  |  |
| Consultation 1 with SWEET study practitioner |  | x  ***(ARM A ONLY)*** |  |  |  |  |  |
| Access to the HT&Me web-app |  | x  ***(ARM A ONLY)*** | | | | |  |
| Text or email motivational nudges |  | x  ***(ARM A ONLY)*** | | | | |  |
| Consultation 2 with SWEET study practitioner |  |  | x  ***(ARM A ONLY)*** |  |  |  |  |
| HT&Me automated text feedback |  | x  ***(ARM A ONLY)*** | x  ***(ARM A ONLY)*** |  |  |  |  |
| Encashment data linkage | x | | | | | | x |
| Survival status  (including date and cause of death where appropriate) |  |  |  | x | x | x | x |
| Disease recurrence assessment |  |  |  | x | x | x | x |
