# Supplementary material for: Improving outcomeS for Women diagnosed with early breast cancer through adhErence to adjuvant Endocrine Therapy (SWEET): study protocol for a pragmatic randomised control trial of a patient-centred intervention to improve adherence to endocrine therapy in early breast cancer
Source: Trials. 2025 Nov 26;26:551. doi: 10.1186/s13063-025-09056-6 (PMC12659038; doi:10.1186/s13063-025-09056-6)
Supplement: Supplementary file 4 — Additional file 4. Consultation 1 (Initial appointment) with SWEET study nurse: Nurse’s Guide. [file 13063_2025_9056_MOESM4_ESM.docx]

#
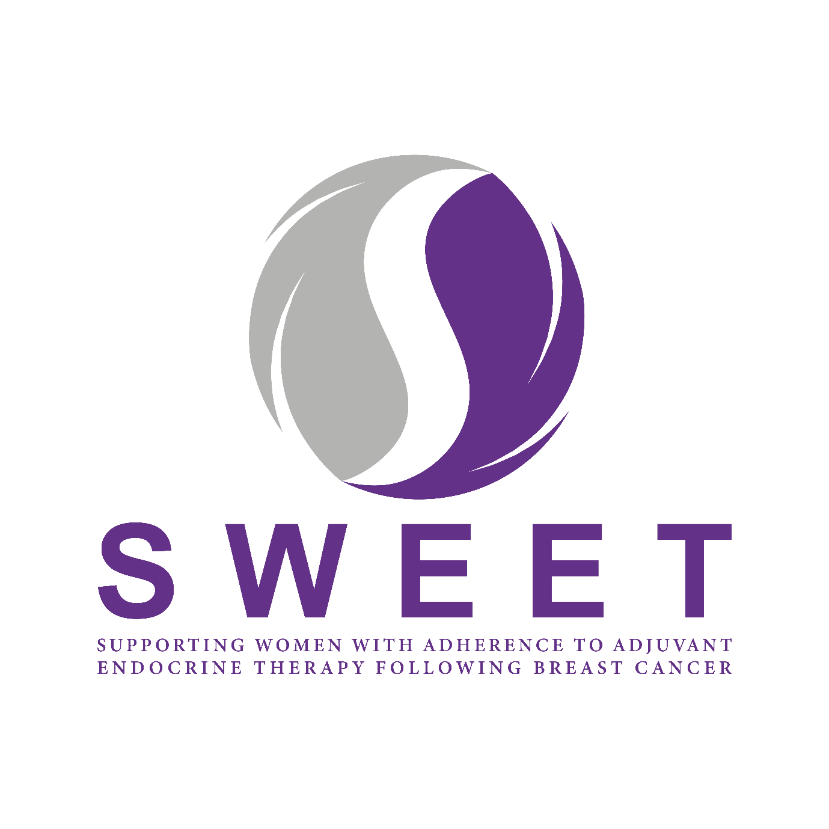
**Consultation 1 (initial appointment) with SWEET study nurse: Nurse’s Guide**

This is intended as a guide for the study nurse(s). The appointment and content will be tailored to the individual patient.

1. **Introduction, checking animation & introducing website (2 mins)**

The purpose of this appointment is to chat with you about the hormone therapy you have recently been prescribed, to make sure you understand why it has been prescribed and why it is important for you to take, to see how you have been getting on with taking it, and to answer any questions or concerns that you may have.

At the end of the session, I will show you the HT&Me website in detail.

Firstly, did you receive the link to short video to watch about HT? [If no, explain will provide the link later]. Did you get the chance to watch it? ***(log response)***

1. **Practical barriers (5 mins)**

Which HT have you been prescribed? Have you started taking your it? How’s that going, are you managing to take it every day?

**Note – the patient may respond to this question to indicate that she doesn’t want to take it or really see the point of taking it. In this case you may find it helpful to refer to the responses in section 3 (beliefs).**

***See example prompts / answers below:***

| **Patient response:** | **HT navigator response:** |
| --- | --- |
| Keep forgetting | - Acknowledge difficulties with starting new treatments, remembering to take it everyday - The best way to remember to take any medication is to try and build a routine and link taking HT with a part of your daily routine - You can find ways to help you do this in the **Taking HT section of HT&Me**, as well as lots of other tips to help you remember. You can also set a daily text or email reminder through the **diary section of HT&Me**. |
| Struggle when my routine isn’t consistent | - This can make it a lot harder - Link taking HT to something that you do every day – like going to bed or brushing your teeth - then you should be reminded to do it even if you are on holiday or at the weekend - You can also set a daily text or email reminder through the **diary section of HT&Me.** |
| Struggle with remembering to collect prescriptions | - Acknowledge difficulties with starting new treatments - Suggest setting an alarm – which you can do in the **diary section of HT&Me.** |
| Other | - Refer to HT&Me / signpost to different resources |

1. **Beliefs about hormone therapy (5-10 mins)**

How do you feel about having to take your hormone therapy every day? Is it something that’s important to you?

***See example prompts / answers below:***

| **Patient response:** | **HT navigator response:** |
| --- | --- |
| Poor understanding of why it’s been prescribed / how it works | - Explain oestrogen is a hormone - Describe benefits of HT / how it works: (may want to refer to animation to show this) - You have been prescribed HT because your cancer was sensitive to oestrogen, this means the cancer cells were fed by oestrogen. - HT works by blocking the effects of oestrogen, so any cancer cells are less likely to grow / spread - Taking tamoxifen blocks the oestrogen from being able to feed the cancer cells. - AIs stop your body from producing oestrogen - HT is a really important part of your cancer treatment – just like surgery/chemo/radiotherapy - **You can find more information in the animation and in the Taking HT section of HT&Me.** |
| Already had enough treatment  or  HT not as important as previous treatments | - Acknowledge that this makes sense - they have already been through enough. - Highlight that HT is **just as important** as chemo/surgery/radio. - HT is the best defence they have now – it works to maintain the hard work of the previous treatment(s) and keep the cancer away. - **You might find it helpful to re-watch the animation or read the information within the Taking HT section of HT&Me.** |
| I’m already doing so much to stay healthy | - Reinforce benefits of a healthy lifestyle in reducing recurrence - There is consistent evidence to show that patients who engage in more physical activity have statistically significant reductions in risk of recurrence and mortality.^[[1]](#footnote-1)^^[[2]](#footnote-2)^^[[3]](#footnote-3)^ - Exercise can also improve emotional wellbeing and reduce fatigue.^[[4]](#footnote-4),^^[[5]](#footnote-5)^ - But highlight that needs to be done **alongside** not instead of HT - HT is scientifically proven to reduce risk of recurrence – at the moment there is evidence that exercise etc is beneficial but none of the evidence is as strong as that for HT - **You can find tips for a maintaining a healthy lifestyle in the Healthy Living, Healthy Mind section of HT&Me.** |
| Don’t think it’s very effective | - Acknowledge concerns – it’s normal to weigh up the pros and cons and think about what is worth it for you - No treatments are 100% effective – so this is normal – We don’t know how effective it will be for you, but we do know that this is the **single best thing you can do right now to reduce the risk of the cancer coming back** - Evidence suggests that across women, those who take HT are less likely to have a recurrence than those who don’t take HT - **You might find it helpful to re-watch the animation or read the information within the Taking HT section of HT&Me** |

1. **Concerns about hormone therapy (5-10 mins)**

Do you have any concerns about hormone therapy? Is there anything that makes you not want to take it?

Do you have any questions?

***See example prompts / answers below:***

| **Patient response:** | **HT navigator response:** |
| --- | --- |
| Side effects – risk of developing | - Acknowledge women do get side effects & the impact they can have - Important to remember that you tend to hear about all the people who aren’t getting on well – people don’t shout as much if they don’t have any side effects - Everyone is different and there is no way to know if you will have any side effects - Even if you had side effects from previous treatment- no reason to think this will be the same here - Lots of side effects lessen over time – and there are many things that can be done to manage them - Go over any concerns regarding specific side effects & provide tips on how these can be managed - **Refer to Dealing with Side Effects in HT&Me.** |
| Side effects – current experience | - Provide support for specific symptoms if relevant - General messaging- Sometimes it can be difficult to disentangle whether symptoms are actually side effects of HT, or side effects from earlier BC treatments or part of the normal ageing process as there is overlap between these. This means that stopping HT will not in all cases mean symptoms disappear. - **Refer to Dealing with Side Effects section of HT&Me** |
| Long lasting impact | - Acknowledge concerns - General advice – whilst it may be scary to take a treatment for a long term, these drugs have been used in thousands of women and the evidence strongly indicates the benefits outweigh any harms. - Deal with any specific concerns – e.g. endometrial cancer (risk of this is low in general population – so even though the risk may be increased with HT it is still not a high risk and is (much) lower than the risk of breast cancer recurrence)^[[6]](#footnote-6)^,^[[7]](#footnote-7)^ - **Refer to About Hormone Therapy section of HT&Me – Questions about the risks and benefits of hormone therapy** |
| General dislike of medicines | - Acknowledge concern – especially when have already been through lots of treatment - Highlight safety testing of HT, importance of doing other things (supplements, healthy lifestyle) alongside rather than instead of HT |
| Don’t like taking medicine for 10 years | - Acknowledge concerns - Reassure this dosage has been safely tested in thousands of women - Each day the body uses up the medicine you have taken – so it doesn’t build up in your body - Highlight how taking it becomes habit – so not something you have to think about |
| Don’t like that it is a reminder of cancer | - Acknowledge difficulties - Try and focus on the benefits the medicine is bringing and reframe into a positive step you are able to take to reduce risk of recurrence - Over time taking HT will become a habit and should be something you are able to think about less - If you are struggling with negative feelings around taking hormone therapy, it may help you to speak to someone about it – signpost to relevant support |
| Other | - Refer to HT&Me / signpost to different resources |

1. **Introduce web app (5 mins)**

Overview of main sections, highlighting where you can set a medication plan, focusing on interactive elements (diary, goals, my personal support).

1. **Close**

Tell the patient you will be back in touch in 3 months, remind them of the HT&Me website, the support available through Breast Cancer Now, and from their clinical team or GP.

Tell patients if they have any questions about the HT&Me website or the study, to get in touch with the research team or the SWEET trials office via email at [htandmesupport@warwick.ac.uk](mailto:htandmesupport@warwick.ac.uk)

1. <https://www.cancerresearchuk.org/about-cancer/causes-of-cancer/physical-activity-and-cancer/what-are-the-benefits-of-exercise> [↑](#footnote-ref-1)
2. <https://pubmed.ncbi.nlm.nih.gov/32239145/> [↑](#footnote-ref-2)
3. <https://www.cancer.gov/news-events/cancer-currents-blog/2020/breast-cancer-survival-exercise> [↑](#footnote-ref-3)
4. https://link.springer.com/article/10.1186/s12885-015-1069-4 [↑](#footnote-ref-4)
5. <https://www.ncbi.nlm.nih.gov/pmc/articles/PMC4622557/> [↑](#footnote-ref-5)
6. https://pubmed.ncbi.nlm.nih.gov/12039943/ [↑](#footnote-ref-6)
7. https://www.ncbi.nlm.nih.gov/pmc/articles/PMC3930906 [↑](#footnote-ref-7)
