# Supplementary material for: Improving outcomeS for Women diagnosed with early breast cancer through adhErence to adjuvant Endocrine Therapy (SWEET): study protocol for a pragmatic randomised control trial of a patient-centred intervention to improve adherence to endocrine therapy in early breast cancer
Source: Trials. 2025 Nov 26;26:551. doi: 10.1186/s13063-025-09056-6 (PMC12659038; doi:10.1186/s13063-025-09056-6)
Supplement: Supplementary file 5 — Additional file 5. Consultation 2 (follow-up appointment) with SWEET study nurse: Nurse’s Guide. [file 13063_2025_9056_MOESM5_ESM.docx]

# **
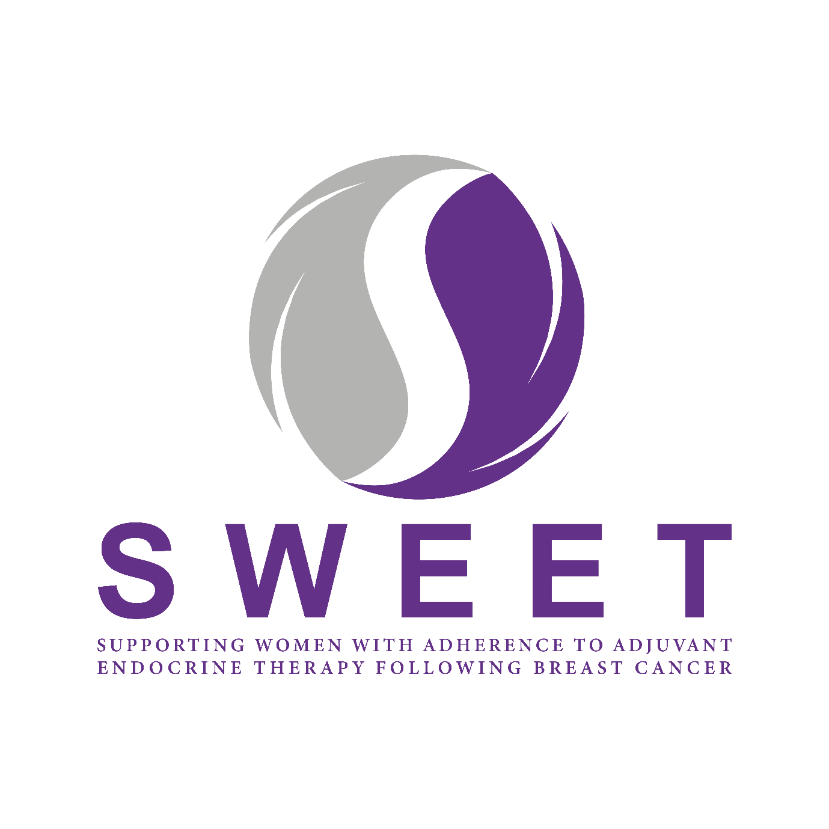
consultation 2 (FOLLOW UP APPOINTMENT) with SWEET study nurse: Nurse’s Guide**

This is intended as a guide for the study nurse(s). The appointment and content will be tailored to the individual patient.

1. **Introduction (2 mins)**

It’s been about 3 months now since we met, and the purpose of this appointment is to check in with you, find out how you have been getting on with taking your hormone therapy, and to answer any questions or concerns that you may have.

Firstly, have you been using the HT&Me website?

***If participant raises any technical issues with website, please refer them to*** [htandmesupport@warwick.ac.uk](mailto:htandmesupport@warwick.ac.uk)

1. **Practical barriers (3 mins)**

Which HT were you prescribed? How’s it going, are you managing to take it every day? Have you managed to get into a good routine? Have you missed any days?

- ***If they are struggling to take it****:* Do you understand why you’ve been prescribed it or / how it works? Would you like me to give you some more information?

***See example prompts / answers below:***

| **Patient response:** | **HT navigator response:** |
| --- | --- |
| Doesn’t really see why it is important | - Explain oestrogen is a hormone - Describe benefits of HT / how it works: (may want to refer to animation which shows this visually) - You have been prescribed HT because your cancer was sensitive to oestrogen, this means the cancer cells were fed by oestrogen. - HT works by blocking the effects of oestrogen, so any cancer cells are less likely to grow / spread - Taking tamoxifen blocks the oestrogen from being able to feed the cancer cells. - AIs stop your body from producing oestrogen - HT is a really important part of your cancer treatment – just like surgery/chemo/radiotherapy - **Refer to Taking HT section of HT&Me** |
| Keep forgetting | - Acknowledge difficulties with starting new treatments, remembering to take it everyday - The best way to remember to take any medication is to try and build a routine and link taking HT with a part of your daily routine - You can find ways to help you do this in the **Taking HT section of HT&Me**, as well as lots of other tips to help you remember. You can also set a daily text or email reminder through the **diary section of HT&Me**. |
| Struggle when my routine isn’t consistent | - This can make it a lot harder - Link taking HT to something that you do every day – like going to bed or brushing your teeth - then you should be reminded to do it even if you are on holiday or at the weekend - You can also set a daily text or email reminder through the **diary section of HT&Me.** |
| Struggle with remembering to collect prescriptions | - Acknowledge difficulties with starting new treatments - Suggest setting an alarm – which you can do in the **diary section of HT&Me.** |
| Other | - Refer to HT&Me / signpost to different resources |

1. **Beliefs about hormone therapy (5 mins)**

How do you feel about having to take your hormone therapy every day? Is it something that’s important to you?

***See example prompts / answers below:***

| **Patient response:** | **HT navigator response:** |
| --- | --- |
| Poor understanding of why it’s been prescribed / how it works | - Explain oestrogen is a hormone - Describe benefits of HT / how it works: (may want to refer to animation to show this) - You have been prescribed HT because your cancer was sensitive to oestrogen, this means the cancer cells were fed by oestrogen. - HT works by blocking the effects of oestrogen, so any cancer cells are less likely to grow / spread - Taking tamoxifen blocks the oestrogen from being able to feed the cancer cells. - AIs stop your body from producing oestrogen - HT is a really important part of your cancer treatment – just like surgery/chemo/radiotherapy - **You can find more information in the animation and in the Taking HT section of HT&Me.** |
| Already had enough treatment  or  HT not as important as previous treatments | - Acknowledge that this makes sense - they have already been through enough. - Highlight that HT is **just as important** as chemo/surgery/radio. - HT is the best defence they have now – it works to maintain the hard work of the previous treatment(s) and keep the cancer away. - **You might find it helpful to re-watch the animation or read the information within the Taking HT section of HT&Me.** |
| I’m already doing so much to stay healthy | - Reinforce benefits of a healthy lifestyle in reducing recurrence - There is consistent evidence to show that patients who engage in more physical activity have statistically significant reductions in risk of recurrence and mortality.^[[1]](#footnote-1)^^[[2]](#footnote-2)^^[[3]](#footnote-3)^ - Exercise can also improve emotional wellbeing and reduce fatigue.^[[4]](#footnote-4),^^[[5]](#footnote-5)^ - But highlight that needs to be done **alongside** not instead of HT - HT is scientifically proven to reduce risk of recurrence – at the moment there is evidence that exercise etc is beneficial but none of the evidence is as strong as that for HT - **You can find tips for a maintaining a healthy lifestyle in the Healthy Living, Healthy Mind section of HT&Me.** |
| Don’t think it’s very effective | - Acknowledge concerns – it’s normal to weigh up the pros and cons and think about what is worth it for you - No treatments are 100% effective – so this is normal – We don’t know how effective it will be for you, but we do know that this is the **single best thing you can do right now to reduce the risk of the cancer coming back** - Evidence suggests that across women, those who take HT are less likely to have a recurrence than those who don’t take HT - **You might find it helpful to re-watch the animation or read the information within the Taking HT section of HT&Me** |

1. **Concerns about hormone therapy (5 mins)**

Do you have any concerns about hormone therapy? Is there anything that makes you not want to take it?

Do you have any questions?

***See example prompts / answers below:***

| **Patient response:** | **HT navigator response:** |
| --- | --- |
| Side effects – risk of developing | - Acknowledge women do get side effects & the impact they can have - Important to remember that you tend to hear about all the people who aren’t getting on well – people don’t shout as much if they don’t have any side effects - Everyone is different and there is no way to know if you will have any side effects - Even if you had side effects from previous treatment- no reason to think this will be the same here - Lots of side effects lessen over time – and there are many things that can be done to manage them - Go over any concerns regarding specific side effects & provide tips on how these can be managed - **Refer to Dealing with Side Effects in HT&Me** |
| Side effects – current experience | - Provide support for specific symptoms if relevant - General messaging- Sometimes it can be difficult to disentangle whether symptoms are actually side effects of HT, or side effects from earlier BC treatments or part of the normal ageing process as there is overlap between these. This means that stopping HT will not in all cases mean symptoms disappear. - Try taking HT at a different time of day to change the time when the side effects appear – may make them easier to cope with - Exercise & physical activity help with almost all side effects - **Refer to Dealing with Side Effects section of HT&Me** |
| Long lasting impact | - Acknowledge concerns - General advice – whilst it may be scary to take a treatment for a long term, these drugs have been used in thousands of women and the evidence strongly indicates the benefits outweigh any harms. - Deal with any specific concerns – e.g. endometrial cancer (risk of this is low in general population – so even though the risk may be increased with HT it is still not a high risk and is (much) lower than the risk of breast cancer recurrence)^[[6]](#footnote-6)^,^[[7]](#footnote-7)^ - **Refer to About Hormone Therapy section of HT&Me – Questions about the risks and benefits of hormone therapy** |
| General dislike of medicines | - Acknowledge concern – especially when have already been through lots of treatment - Highlight safety testing of HT, importance of doing other things (supplements, healthy lifestyle) alongside rather than instead of HT |
| Don’t like taking medicine for 5-10 years | - Acknowledge concerns - Reassure this dosage has been safely tested in thousands of women - Each day the body uses up the medicine you have taken – so it doesn’t build up in your body - Highlight how taking it becomes habit – so not something you have to think about |
| Don’t like that it is a reminder of cancer | - Acknowledge difficulties - Try and focus on the benefits the medicine is bringing and reframe into a positive step you are able to take to reduce risk of recurrence - Over time taking HT will become a habit and should be something you are able to think about less - If you are struggling with negative feelings around taking hormone therapy, it may help you to speak to someone about it – signpost to relevant support |
| Other | - Refer to HT&Me / signpost to different resources |

1. **Close –**

End consultation. Remind participant of the help and tips in the HT&Me website, and that if they are struggling they can speak to their breast cancer team, GP or pharmacist, and can also access the support available through Breast Cancer Now.

Tell patients if they have any questions about the HT&Me website or the study, to get in touch with the research team or the SWEET trials office via email at [htandmesupport@warwick.ac.uk](mailto:htandmesupport@warwick.ac.uk)

The next contact through the intervention will be when they receive their follow up questionnaires.

***Extra support for specific side effects:***

Below are some helpful tips for managing some of the common side effects. You can refer women to the HT&Me website to find out more about any of these suggestions.

| ***Hot flushes /night sweats*** | Practical tips:   - Keep surroundings cool, loose fitting clothes, light layers. - Carry an electric fan - Avoid smoking and look for other triggers like caffeine, alcohol or spicy foods - Try cooling products like gel pillows or neck ties - Try physical activity - Complementary therapies   Change the way you think about hot flushes / night sweats (using CBT approaches)   - Notice what goes through your mind when you have a hot flush and try to challenge any negative thoughts, developing calmer, more supportive responses instead - Try paced breathing   Speak to clinical team or GP about trying medication that helps to control hot flushes*.* |
| --- | --- |
| ***Joint aches & pains*** | - Try taking HT at different time of the day - Speak to GP about what pain medication may be available. - Use warm or cold packs to ease pain. - Take warm baths - Keep active – try walking, swimming, yoga – speak to GP if worried about what exercise you can do. Regular exercise can strengthen the muscles around joints which helps to keep them flexible and reduce pain - Acupuncture |
| ***Fatigue*** | - Keep a diary of fatigue (use HT&Me diary) - Plan ahead – plan days around when you feel most energised - Keep active – try walking, swimming, yoga – speak to GP if worried about what exercise you can do. Regular exercise can reduce physical fatigue, painful joints and muscle aches as well as boosting mood and helping with sleep - Eat a healthy balanced diet - Try to get a good night sleep by having a regular sleep routine where you wake up and go to bed at the same time each day. For some, taking naps in the day may help with sleeping at night, but for others it can make it more difficult to fall asleep or stay asleep. - Talk to friends and family about fatigue - Try to manage any feelings of being low or anxious that may make you feel more fatigued - Complementary therapies such as yoga, meditation and acupuncture can be helpful in managing mood   Change the way you think about fatigue (using CBT approaches)   - Notice what goes through your mind when you feel fatigued and try to challenge any negative thoughts, developing calmer, more supportive responses instead - Try to avoid boom and bust: extremes of rest and activity can make your fatigue feel worse |
| ***Sexual concerns*** | Loss of desire and arousal   - Try sensate focus as a way of focusing on your own or your partner’s touch. The aim is for each person to let go of their expectations and judgements about how something ‘should be’ or ‘should feel’, and instead just enjoy the touch.   Sharing thoughts and feelings   - Being able to talk about your needs and struggles with your partner can help you to feel safe and closer to your partner, and it can be the first step on the way to become more sensual, erotic and then sexual. |
| ***Weight changes*** | Practical tips:   - Eat a heathy balanced diet - Keep active – regular physical activity and keeping active in your daily life can help you to maintain a healthy weight. These could be simple changes that can be introduced into your life to increase your levels of activity e.g., doing some gardening or getting off the bus a stop earlier than needed.   Practical tips to maintain these changes:   - Keep a routine – eating meals at roughly the same time every day can help to reduce snacking and unplanned meals - Make plans – making a clear and specific plan to do something increases our chances of making lifestyle changes. - Make small and gradual changes - Cut down rather than cut out – lifestyle changes that last are often about balance. Cutting out all your favourite foods and drinks often makes us want them more and change is therefore unlikely to be maintained over time. |
| ***Mood changes*** | Managing mood changes and irritability:   - Talk to others about how you are feeling - Be active and exercise regularly - Take care of your body – maintain a healthy diet and try to get enough sleep - Make time for yourself – take care of your mind and be kind to yourself. Keep up with hobbies and things you enjoy whether this be listening to music, having a bath or spending time with pets or family. - Meditation or mindfulness may help you to relax.   Challenging negative thinking:   - Challenge your negative thoughts and try to reach a place of acceptance. Try coming up with self-supporting responses to automatic thinking. For example, notice a thought e.g., ‘*This will never end’* and challenge that with a self-supporting response e.g., ‘*This is just an intense moment, it will pass, and I know that with time these settle for most women’.*   If you notice that most or all of the time, for at least two weeks, you are feeling low in mood, are not finding your usual pleasures or interests in everyday life, are withdrawing or being very irritable, are close to tears and easily overwhelmed or are constantly thinking the worst we strongly recommend that you tell a healthcare professional that you trust (maybe your GP, cancer nurse specialist, or consultant) |
| ***Vaginal dryness and pain*** | Lubricants and vaginal moisturisers   - Intimate lubricants and moisturisers (which can be used inside the body) can help with vaginal dryness and irritation. These are not just for having sex – can be used every day or every few days to improve comfort. These can be bought over the counter, or on the web, or you can get a prescription from your GP. |
| ***Sleep problems*** | Practical tips:   - Try to manage any physical symptoms (such as pain). This could be through over-the-counter or prescribed painkillers before going to sleep - Try out other techniques that can be used to soothe or relieve symptoms around bedtime such as relaxation exercises, massage or a warm bath. - Try taking your hormone therapy at a different time during the day if you think your symptom (e.g., joint pain) is linked to your hormone therapy - Make sure your bedroom environment is the best possible for a good night sleep (e.g., quiet, dark, supportive and comfortable bed) - Create a good sleep routine – e.g., try to create a buffer zone to put the day to sleep. - Set realistic sleep goals that match your own sleep needs – not everyone needs 8 hours - Limit caffeine, nicotine and alcohol before bed - Keep active   Change the way you think and feel about sleep (using a CBT approach)   - Problems with sleep can be stressful and being anxious and stressed can make it more difficult for you to fall or stay asleep. So, combatting stress could help to improve your sleep. Try learning calmer more neutral responses to feelings of stress to help you to feel more in control and more able to cope. |
| ***Other side-effects*** | **Memory**  Practical tips:   - Keep a diary or calendar to remember activities and appointments - Make ‘to do’ lists of important tasks - Break down phone numbers or passwords into segments - Use secure functions on personal computers to automatically save and store passwords - Mind games such as crosswords or Sudoku can help with memory and concentration - Meditation and mindfulness can help with memory and concentration.   **Feeling sick**   - Taking your HT tablet at night may help you to feel less nauseous - If nausea does not improve, speak to practice nurse or GP. They may be able to give your further advice or drugs to help   **Skin changes**   - If changes to skin, hair and nails persist or get worse speak to your practice nurse or GP. - If you get a severe skin rash it is very important to contact your GP straight away. - Buy a decent moisturizer or emollient cream (ideally unperfumed) over the counter – doesn’t have to be expensive (e.g. E45)   **Headaches**   - Take usual painkillers - Talk to practice nurse or GP if keep getting headaches regularly |

1. <https://www.cancerresearchuk.org/about-cancer/causes-of-cancer/physical-activity-and-cancer/what-are-the-benefits-of-exercise> [↑](#footnote-ref-1)
2. <https://pubmed.ncbi.nlm.nih.gov/32239145/> [↑](#footnote-ref-2)
3. <https://www.cancer.gov/news-events/cancer-currents-blog/2020/breast-cancer-survival-exercise> [↑](#footnote-ref-3)
4. https://link.springer.com/article/10.1186/s12885-015-1069-4 [↑](#footnote-ref-4)
5. <https://www.ncbi.nlm.nih.gov/pmc/articles/PMC4622557/> [↑](#footnote-ref-5)
6. https://pubmed.ncbi.nlm.nih.gov/12039943/ [↑](#footnote-ref-6)
7. https://www.ncbi.nlm.nih.gov/pmc/articles/PMC3930906 [↑](#footnote-ref-7)
