# Supplementary material for: Improving outcomeS for Women diagnosed with early breast cancer through adhErence to adjuvant Endocrine Therapy (SWEET): study protocol for a pragmatic randomised control trial of a patient-centred intervention to improve adherence to endocrine therapy in early breast cancer
Source: Trials. 2025 Nov 26;26:551. doi: 10.1186/s13063-025-09056-6 (PMC12659038; doi:10.1186/s13063-025-09056-6)
Supplement: Supplementary file 6 — Additional file 6. Template consent form. [file 13063_2025_9056_MOESM6_ESM.docx]

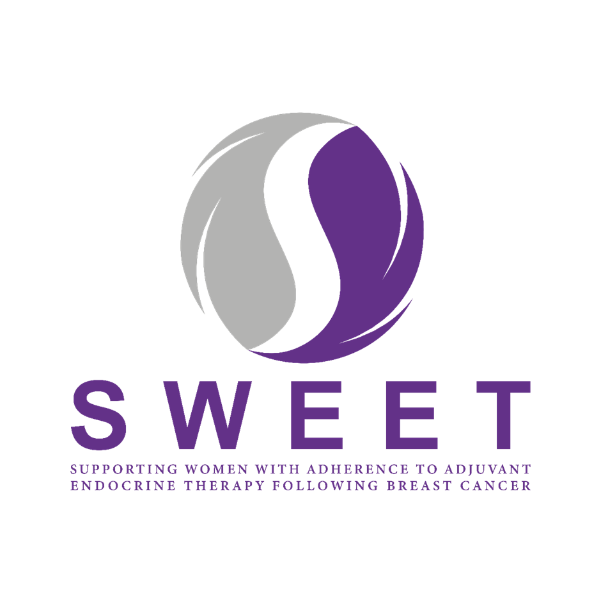


Participant Informed Consent Form

**Title of Project:** Improving outcome**S** for **W**omen diagnosed with early breast cancer through adh**E**rence to adjuvant **E**ndocrine **T**herapy **(SWEET)**

**Name of Researcher:**

**Centre Name/Number: Trial Number**:

**Participants name:____________________________________________________________**

*This consent form applies to patients being consented either In Person or Remotely (Verbal)*

*If In Person: The local Principal Investigator or designee or study team researcher obtaining consent must read out each point on the consent form individually. The patient must initial each box to confirm that they agree to each point and must sign section A2*

*If Remote Verbal Consent: The local Principal Investigator or designee or study team researcher obtaining consent must read out each point on the consent form individually and must initial each box to confirm the patient agrees to each point. The process should be overseen by a witness. The consent form must be signed by the local Principal Investigator or designee and countersigned by the witness.(See B1 & B2)*

***Initial each box***

| 1. | I confirm that I have read and understand the SWEET information sheet Version.........., Dated............ for the above study. I have had the opportunity to consider the information, ask questions and have had these questions answered satisfactorily. |  | |
| --- | --- | --- | --- |
| 2. | I understand that my participation is voluntary and that I am free to withdraw at any time, without giving any reason, without my medical care or legal rights being affected. |  | |
| 3. | I understand that data collected during the study may be looked at by authorised individuals from Warwick Clinical Trials Unit (WCTU) and the Sponsor (The Newcastle-upon-Tyne Hospitals NHS Foundation Trust), the research team including collaborators from University College London and Oxford Brookes, and regulatory authorities or by the local NHS recruiting trust, where it is relevant to my taking part in this research. I give permission for these individuals to have access to my records. |  | |
| 4. | I understand that personal data (including name, ethnicity, address, telephone number, email address and [NHS number or insert equivalent for devolved nation]) will be stored on a secure database so I can be contacted by individuals authorised to do so during the study. I understand that the research team, including nurses from Breast Cancer Now (where applicable) will be able to access this data to contact me about appointments. |  | |
| 5. | I agree to my General Practitioner being informed of my participation in the study. |  | |
| 6. | I understand that members of my hospital care team may request data about my health from my hospital and GP records, where it is relevant to my taking part in this research. |  | |
| 7. | I understand that the information collected about me may be used to support other research in the future and anonymised or coded data (pseudonymised data) may be shared with other researchers. |  | |
| 8. | I understand that the information will be stored securely and only used for medical research purposes and that I will not be identified in any way in the analysis and reporting of the results. |  | |
| 9. | I understand that some details will be taken from my hospital records to link with [NHS England or insert equivalent NHS database for devolved nation], and/or my GP records to allow information regarding my breast cancer related treatment to be studied. I understand that the linked data will be coded (pseudonymised) and will have my personal identifying information removed. |  | |
| 10. | I understand that my appointment with the SWEET study Nurse/Practitioner may be audio-recorded and listened to by the research team for quality control purposes. |  | |
| 11. | I understand that I may be sent text messages and/or emails on behalf of the SWEET team for the purposes of the study. A third-party service may be used to send text/ or email messages or to enter questionnaire data related to the study and that my contact details provided will facilitate this. |  | |
| 12. | I agree to data being collected for up to 15 years for the purpose of long-term follow-up. I understand this will mostly involve taking information from my hospital records, GP records, or NHS databases, but that I may be contacted and asked some questions. |  | |
| 13. | I agree to take part in the above study. |  | |
| **OPTIONAL CONSENT POINTS** *(please initial either yes or no)* | | Yes | No |
| 14. | **OPTIONAL**  I would like to receive a copy of the results when they are available. |  |  |
| 15. | **OPTIONAL**  I agree to take part in an interview about my experience of the trial, and understand these interviews will be audio recorded |  |  |
| 16. | **OPTIONAL**  I agree to be contacted about future research studies in this area |  |  |

| **Consent Type – please Select 1 Option Only *(to be completed by investigator taking consent)*** | Yes |
| --- | --- |
| Informed Consent- In Person *(Please proceed to A1 and A2)* |  |
| Remote Verbal Consent *(Please proceed to B1 and B2)* |  |

| **A1. *To be completed by the person taking consent.*** | | |
| --- | --- | --- |
| Name of person taking consent (print):  __________________________________ | Signature:  _________________________________ | Date signed:  ______________________ |
| **A2. To be completed by the Patient** |  |  |
| Patient Name (print):  __________________________________ | Signature:  _________________________________ | Date signed:  ______________________ |

| **B1. Investigator Statement and Signature.**  ***To be completed by the person taking consent for Remote Verbal only.*** | | |
| --- | --- | --- |
| I have discussed this clinical research study with the participant. I believe that I have fully informed the participant of the nature of this study and the possible benefits and risks of taking part. I believe the participant has understood this explanation. | | |
| Name (print):  __________________________________ | Signature:  _________________________________ | Date signed:  ______________________ |
| **B2. Witness signature** |  |  |
| Name (print):  __________________________________ | Signature:  _________________________________ | Date signed:  ______________________ |

**NB: Three copies should be made: Original to be retained in Investigator Site File, 1 copy for patient, 2nd copy for medical notes.**
